# Lupus susceptibility gene *Esrrg* modulates regulatory T cells through mitochondrial metabolism

**DOI:** 10.1172/jci.insight.143540

**Published:** 2021-07-22

**Authors:** Wei Li, Minghao Gong, Yuk Pheel Park, Ahmed S. Elshikha, Seung-Chul Choi, Josephine Brown, Nathalie Kanda, Wen-I Yeh, Leeana Peters, Anton A. Titov, Xiangyu Teng, Todd M. Brusko, Laurence Morel

**Affiliations:** 1 Department of Pathology, Immunology and Laboratory Medicine, College of Medicine, University of Florida, Gainesville, Florida, USA.; 2 Department of Pharmaceutics, Zagazig University, Zagazig, Egypt.

**Keywords:** Autoimmunity, Lupus, Mitochondria, T cells

## Abstract

Estrogen-related receptor γ (*Esrrg*) is a murine lupus susceptibility gene associated with T cell activation. Here, we report that *Esrrg* controls Tregs through mitochondria homeostasis. *Esrrg* deficiency impaired the maintenance and function of Tregs, leading to global T cell activation and autoimmunity in aged mice. Further, *Esrrg*-deficient Tregs presented an impaired differentiation into follicular Tregs that enhanced follicular helper T cells’ responses. Mechanistically, *Esrrg*-deficient Tregs presented with dysregulated mitochondria with decreased oxygen consumption as well as ATP and NAD^+^ production. In addition, *Esrrg*-deficient Tregs exhibited decreased phosphatidylinositol and TGF-β signaling pathways and increased mTOR complex 1 activation. We found that the expression of human *ESRRG*, which is high in Tregs, was lower in CD4^+^ T cells from patients with lupus than in healthy controls. Finally, knocking down *ESRRG* in Jurkat T cells decreased their metabolism. Together, our results reveal a critical role of *Esrrg* in the maintenance and metabolism of Tregs, which may provide a genetic link between lupus pathogenesis and mitochondrial dysfunction in T cells.

## Graphical abstract

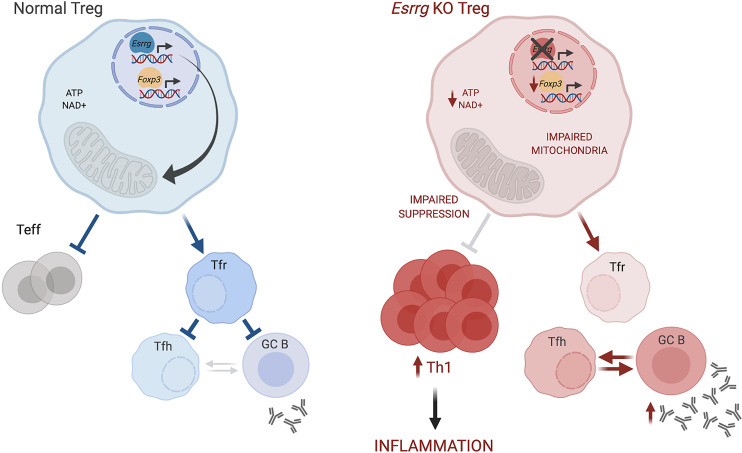

## Introduction

Tregs are a CD4^+^ T cell subpopulation that is essential to maintain immune homeostasis and prevent autoimmune diseases (1). Tregs are characterized by surface expression of CD25, the high-affinity subunit α of the interleukin-2 (IL-2) receptor (2), along with expression of the lineage-defining transcription factor FOXP3 (3). The constitutive expression of CD25 on the surface of Tregs is accompanied by a dependence on exogenous IL-2 for Treg maintenance and metabolic fitness (4). The thymus is the primary source of Tregs (thymus Tregs, tTregs), which express neuropilin 1 (Nrp1) and Helios (5, 6). Tregs can also differentiate from naive CD4^+^ T cells in peripheral tissues (peripheral Tregs, pTregs), or they can be induced in vitro by the stimulation of the T cell receptor in the presence of TGF-β and IL-2 (induced Tregs, iTregs) (5, 7). In patients with systemic lupus erythematosus (SLE) as well as in mouse models of the disease, multiple defects have been reported in Treg populations (8). Patients with SLE present an expanded population of dysfunctional Tregs that express low levels of CD25 (9) and FOXP3 (10, 11). Follicular helper T (Tfh) cells, a CD4^+^ T cell subset specialized to help germinal center (GC) B cells for high-affinity antibody production, are found in high numbers in patients with SLE (12) and in lupus-prone mice (13), in correlation with disease activity. Tfh cells are regulated by follicular regulatory T (Tfr) cells, which differentiate from tTregs in GCs by acquiring some of the BCL6 transcription repressor–driven (BCL6-driven) Tfh cell transcriptional program (14, 15). Tfr cells inhibit Tfh expansion and function (16). Directly relevant to lupus, deletion of Tfr cells in mice increases the production of autoantibodies (17), and the Tfh/Tfr ratio correlates with disease activity in patients with SLE (18).

Increasing evidence suggests that metabolic regulation is essential for Treg homeostasis, function, and stability (19). Effector T (Teff) cells utilize mTOR complex 1–driven (mTORC1-driven) glycolysis (20). Glycolysis is also required for Treg activation and proliferation (21). However, high levels of mTORC1 activation prevent FOXP3 expression, and glycolysis impairs Treg function (22, 23). Accordingly, Tregs deficient in serine/threonine protein phosphatase PP2A, a negative regulator of mTORC1, show enhanced mTORC1 signaling and glycolysis and defective suppressive functions (24). In contrast, mTORC1 deficiency in Tregs leads to fatal autoimmunity, and mTORC2 deficiency reduces the frequency of Tregs (25). Interestingly, high levels of mTORC2 were found in impaired mTORC1-deficient or phosphatase and tensin homolog–deficient (PTEN-deficient) Tregs (26). These results indicate that either the lack or the overactivation of mTOR impairs Treg stability and function. Tregs also express high levels of activated AMPK, which inhibits mTORC1, and enhances fatty acid oxidation (27). Deletion of the AMPK upstream sensor *Lkb1* in Tregs results in fatal inflammatory disease independently from the AMPK/mTORC1 pathway (28–30), suggesting an essential but complex role of metabolic regulators in Tregs. Tregs rely on oxidative phosphorylation (OXPHOS) to maintain their suppressive function, which places mitochondria at the core of Treg metabolism. Deletion of the *Uqcrsf1* gene, a subunit of mitochondrial complex III, in Tregs leads to a lethal inflammatory and autoimmune disease in mice (31). Similarly, disruption of mitochondrial transcription factor A (*Tfam*), a nuclear gene crucial for mitochondrial respiration and DNA replication, impairs Treg maintenance and function, resulting in severe systemic inflammation in mice (32). Thus, mitochondrial homeostasis and respiration are essential to maintain Treg function.

We have identified 3 major lupus susceptibility loci in the NZM2410 mouse model that are necessary and sufficient in combination to induce lupus nephritis on a non-autoimmune B6 background in the *B6.Sle1.Sle2.Sle3* triple-congenic strain (33). *Sle1* is necessary for the development of systemic autoimmunity (34), by intrinsically promoting the production of activated, autoreactive CD4^+^ T cells (35). *Sle1* contains a cluster of susceptibility loci (36), among which we have identified *Sle1c2* and its unique candidate gene, estrogen-related receptor γ (*Esrrg*) (37). The lupus susceptibility allele of *Esrrg* corresponds to a lower expression that correlates with CD4^+^ T cell activation and defective Tregs (37). *Esrrg* encodes an orphan nuclear receptor, ERRγ, which regulates oxidative metabolism and mitochondrial function (38). Germline deletion of *Esrrg* results in the fatal inability of the neonatal heart to transition to OXPHOS (39). Tissue-specific deletions have shown that *Esrrg* is essential to maintain mitochondrial metabolism by driving a transcriptional network that activates OXPHOS, the electron transport chain, and ATP production in neurons (40), brown adipose tissue (41), pancreatic β cells (42), chondrocytes (43), and gastric cancer cells (44). *Esrrg* has also been involved in other cellular pathways, such as the suppression of NF-κB transcriptional activity in osteoclasts (43). However, the role of *Esrrg* in modulating the immune system, and T cells in particular, has not been characterized.

Here we show that Tregs with an *Esrrg* conditional deletion (*Esrrg-*cKO) exhibited an impaired peripheral homeostasis and suppressive function, which led to a general T cell activation and the production of autoantibodies in aged mice. Further, *Esrrg-*cKO mice displayed dysregulated Tfh and Tfr responses at steady state and to T-dependent immunization, associated with an impaired differentiation of Tregs into Tfr cells. Mechanistically, *Esrrg-*cKO Tregs exhibited defective mitochondrial functions, coupled with altered expression of genes involved in mitochondrial and Treg programs. Finally, using CRISPR/Cas9-mediated editing, we demonstrate that *ESRRG* deficiency in Jurkat T cells impaired their metabolism. We also show that *ESRRG* was highly expressed in human Tregs and that patients with SLE presented a lower *ESRRG* expression in total CD4^+^ T cells than healthy controls. We propose that the lupus susceptibility gene *Esrrg* is required to maintain suppressive Tregs, and a low expression of *Esrrg* in Tregs may contribute to autoimmune pathogenesis.

## Results

### Esrrg deficiency in Tregs leads to CD4^+^ T cell activation and autoimmunity in aged mice.

**Figure 1:**
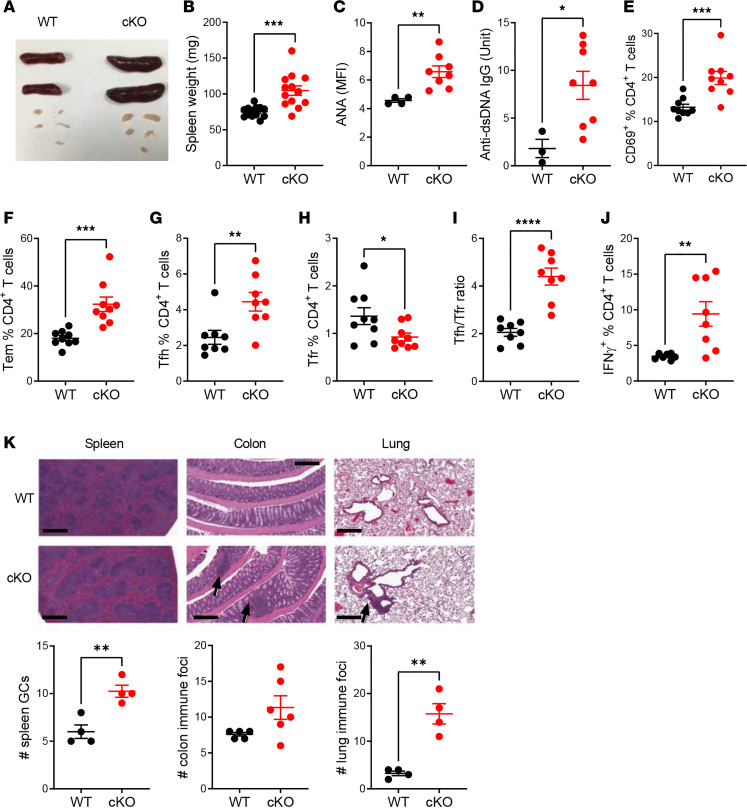
*Esrrg* deficiency in Tregs leads to CD4^+^ T cell activation and autoimmunity in aged mice. (**A**) Representative spleens and mLNs from B6N.*Foxp3*^YFP-Cre^ (WT, shown in black) and B6N.*Esrrg*^fl/fl^ *Foxp3*^YFP-Cre^ (cKO, shown in red) mice. (**B**) Spleen weight. Serum anti-nuclear autoantibodies (ANAs) quantified as mean fluorescence intensity (MFI), with a value of 4 corresponding to background levels (**C**), and anti-dsDNA IgG (**D**). Percentage of CD69^+^ T cells (**E**), CD44^+^CD62L^–^ Tem cells (**F**), Bcl6^+^PD-1^+^CXCR5^+^FOXP3^–^ Tfh cells (**G**), and Bcl6^+^PD-1^+^CXCR5^+^FOXP3^+^ Tfr cells (**H**) in total CD4^+^ T cells. PD-1, programmed cell death protein 1. (**I**) Tfh/Tfr ratio. (**K**) Representative H&E staining of spleen (original magnification, 4×; scale bar: 600 μm), colon, and lung (original magnification, 8×; scale bar: 300 μm) sections from WT and cKO mice, with corresponding quantitation of the number of GCs in the spleen and immune foci (arrows) in the colon and lung per section. Data were obtained from 2- to 3-month-old (**A**, **B**, and **E**–**J**) and 10- to 13-month-old (**D**, **E**, and **K**) mice. Graphs show mean ± SEM with each symbol representing a mouse (*n* = 4 — 13). Unpaired 2-tailed *t* tests, *P < 0.05, **P < 0.01, ***P < 0.001, ****P < 0.0001.

To test the role of *Esrrg* in the development and function of Tregs, we crossed mice containing a *LoxP*-flanked exon 6 of *Esrrg* (Supplemental Figure 1; supplemental material available online with this article; https://doi.org/10.1172/jci.insight.143540DS1) with *Foxp3*^YFP-Cre^ mice to generate mice with a conditional deletion of *Esrrg* in Tregs (B6N.*Foxp3*^YFP-Cre^ *Esrrg*^fl/fl^, *Esrrg-*cKO). B6N.*Foxp3*^YFP-Cre^ mice were used as controls. Deletion of *Esrrg* in Tregs was confirmed by PCR at the genomic and gene expression levels (Supplemental Figure 2, A and B). *Esrrg-*cKO mice exhibited conspicuous signs of inflammation. Their mesenteric lymph nodes (mLNs) and spleens were already enlarged by 2–3 months of age (Figure 1, A and B), and ANAs and anti-dsDNA IgG production were found in the serum of aged *Esrrg-*cKO mice (Figure 1, C and D). Young mice showed an increased frequency of splenic CD4^+^ T cells with an activated phenotype, such as CD69 expression (Figure 1E), and a CD44^+^CD62L^–^ effector memory (Tem) phenotype (Figure 1F). Moreover, *Esrrg-*cKO mice displayed an elevated frequency of Bcl6^+^PD-1^+^CXCR5^+^FOXP3^–^ Tfh cells and a decreased frequency of Bcl6^+^PD-1^+^CXCR5^+^FOXP3^+^ Tfr cells, which resulted in an increased Tfh/Tfr ratio (Figure 1, G–I). Due to the elevated number of total splenocytes, the numbers of each effector subset were dramatically increased in *Esrrg-*cKO mice, while the numbers of naive T (Tn) and Tfr cells were similar between strains (Supplemental Figure 2, C–E). In addition, CD4^+^ T cells from *Esrrg-*cKO mice produced more IFN-γ (Figure 1J and Supplemental Figure 2F), while the frequency of IL-17A^+^ T cells showed no difference between groups (Supplemental Figure 2G). Older *Esrrg-*cKO mice presented a higher number of GCs in the spleen and developed a higher number of immune infiltrate foci in the lung with a similar trend in the colon (Figure 1K). Overall, these results suggest that *Esrrg* deficiency in Tregs induces a global CD4*^+^* T cell activation that leads to the production of autoantibodies in aged mice.

### Esrrg maintains the suppressive function of Tregs.

**Figure 2:**
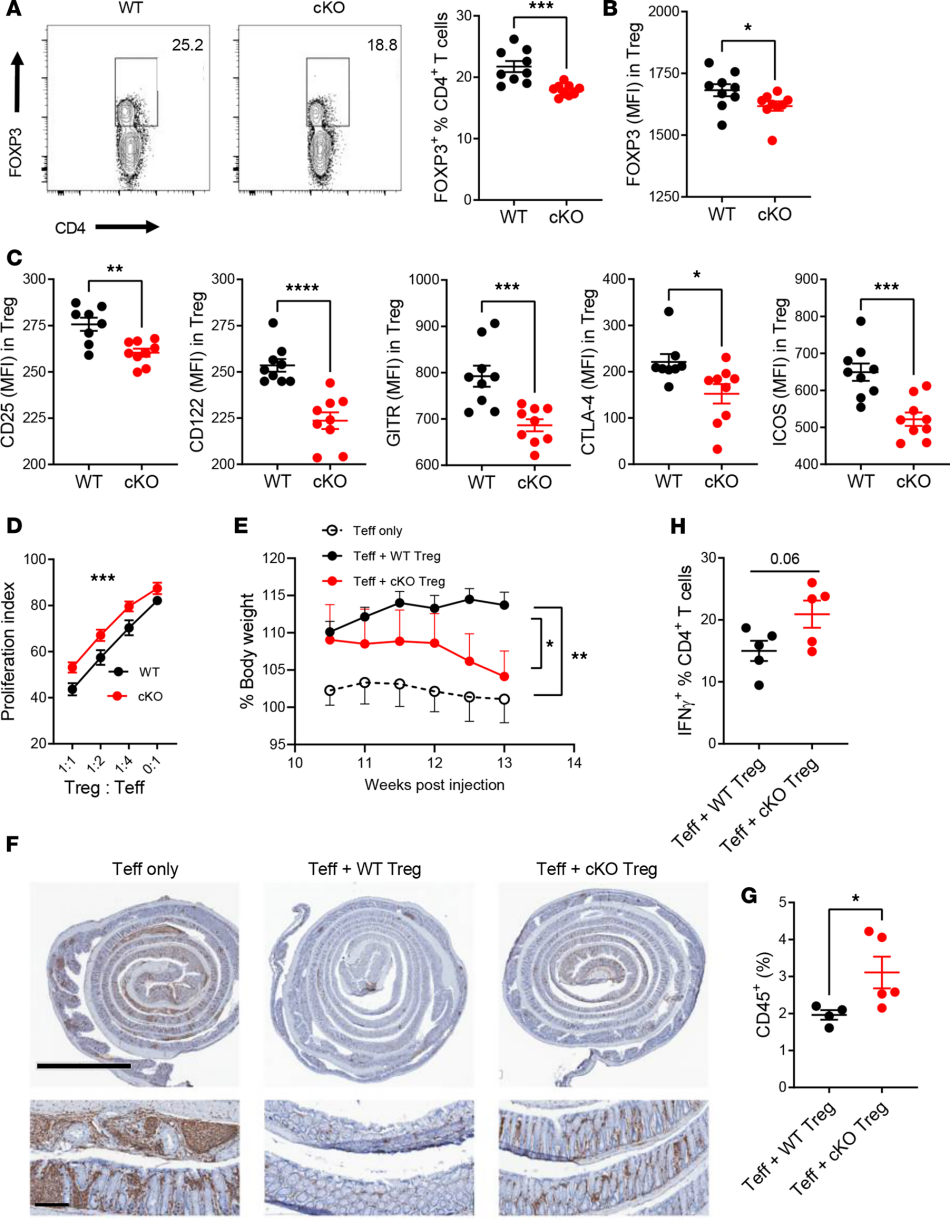
*Esrrg* is required to maintain Treg suppressive function. (**A**) Representative CD4^+^-gated FACS plots showing splenic FOXP3^+^ Tregs from 2- to 3-month-old B6N.*FOXP3*^YFP-Cre^ (WT) and B6N.*FOXP3*^YFP-Cre^ *Esrrg*^fl/fl^ (cKO) mice. FOXP3 (**B**), CD25, CD122, GITR, CTLA-4, and ICOS (**C**) expression measured as MFI in WT and cKO Tregs. (**D**) Teff cell proliferation index with WT and cKO Tregs compared by 2-way ANOVA. Results were combined from 3 assays with 3 mice per group. (**E**–**G**) Treg suppressive assay in the colitis model (*n* = 4–5 per group). (**E**) Body weight change starting at 10.5 weeks after T cell transfer. Statistical comparisons were made between terminal values. (**F**) Representative colons of mice that received Teff cells only or Teff cells with WT/cKO Tregs, stained for CD45^+^ cells (brown). Top row original magnification, 1× (scale bar: 3 mm); bottom row original magnification, 6× (scale bar: 400 μm). (**G**) Quantification of CD45^+^ infiltrates relative to the total cell numbers in the entire section. (**H**) Percentage of IFN-γ^+^ cells in splenic CD4^+^ T cells of the recipient mice. Data are shown as mean ± SEM, *n* = 4–9. Unpaired 2-tailed *t* tests, *P < 0.05, **P < 0.01, ***P < 0.001, ****P < 0.0001.

The frequencies of Tregs (Figure 2A) and HELIOS^+^ Tregs (Supplemental Figure 3A) were significantly reduced in the spleens of *Esrrg-*cKO mice. HELIOS is a marker of tTregs with an activated phenotype and a higher suppressive capacity (45, 46). However, *Esrrg* deficiency did not affect the frequency of tTregs, where the frequency of both NRP-1^+^ tTregs and peripherally generated NRP-1^–^ pTregs was similar between the 2 groups (Supplemental Figure 3B). A similar frequency of splenic Ki-67^+^ and annexin V^+^ Tregs was found in the 2 groups (Supplemental Figure 3, C and D). These results suggest that *Esrrg* expression promotes the maintenance of tTregs in the periphery and that the reduced frequency of *Esrrg-*cKO Tregs in the periphery may not be due to either decreased homeostatic proliferation or increased apoptosis.

*Esrrg* deficiency also impaired the expression of markers associated with the Treg program. The expression of FOXP3 itself was reduced in *Esrrg-*cKO Tregs (Figure 2B). *Esrrg-*cKO Tregs also showed a decreased expression of the IL-2 receptor subunits α (CD25) and β (CD122), glucocorticoid-induced tumor necrosis factor receptor-related protein (GITR), cytotoxic T lymphocyte-associated protein 4 (CTLA-4), inducible T cell costimulatory molecule (ICOS), and integrin αE (CD103), which defines tissue-resident Tregs (47) (Figure 2C and Supplemental Figure 3, E and F). Since each of these molecules is associated with Treg suppressive function, these results suggest that *Esrrg* deficiency impairs Treg function.

To support this hypothesis, we assessed the suppressive function of *Esrrg-*cKO iTregs. Consistent with reduced expression of functional Treg markers, Teff cells proliferated more when cocultured with *Esrrg-*cKO Tregs than with control Tregs (Figure 2D and Supplemental Figure 3G). Next, we tested the Treg suppressive function in vivo in a Teff cell transfer-induced colitis model (Supplemental Figure 3H). As expected, control Tregs prevented the development of colitis in recipient mice, but *Esrrg-*cKO Treg recipients showed a decreased body weight (Figure 2E) as well as an increased infiltration of CD45^+^ immune cells in the colon (Figure 2, F and G). *Esrrg-*cKO Treg recipients also showed a trend of increased IFN-γ production in splenic CD4^+^ T cells (Figure 2H). These results suggest that *Esrrg-*cKO Tregs had an impaired suppressive capacity. IL-10 is one of the molecules by which Tregs exert their suppressive functions. However, the frequency of IL-10–producing Tregs was similar between *Esrrg-*cKO and control mice (Supplemental Figure 3I), indicating the loss of suppressive function in *Esrrg-*cKO Tregs is IL-10 independent.

Finally, we evaluated the effect of *Esrrg* deficiency on Treg differentiation in vitro. A reduced frequency of iTregs polarized from CD4^+^CD25^–^ T cells was observed in *Esrrg-*cKO as compared with control T cells (Supplemental Figure 4A), with highly decreased FOXP3 expression in the *Esrrg-*cKO iTregs (Supplemental Figure 4B). A similar result was obtained with iTreg polarization from CD4^+^CD25^–^ T cells in which *Esrrg* was silenced in B6N T cells with small interfering RNA (Supplemental Figure 4C). These results indicate that *Esrrg* expression enhances iTreg differentiation. Overall, these experiments suggest that ERRγ promotes the peripheral maintenance of Tregs and their suppressive capacity.

### Esrrg-cKO mice show dysregulated Tfh and Tfr responses to immunization.

**Figure 3:**
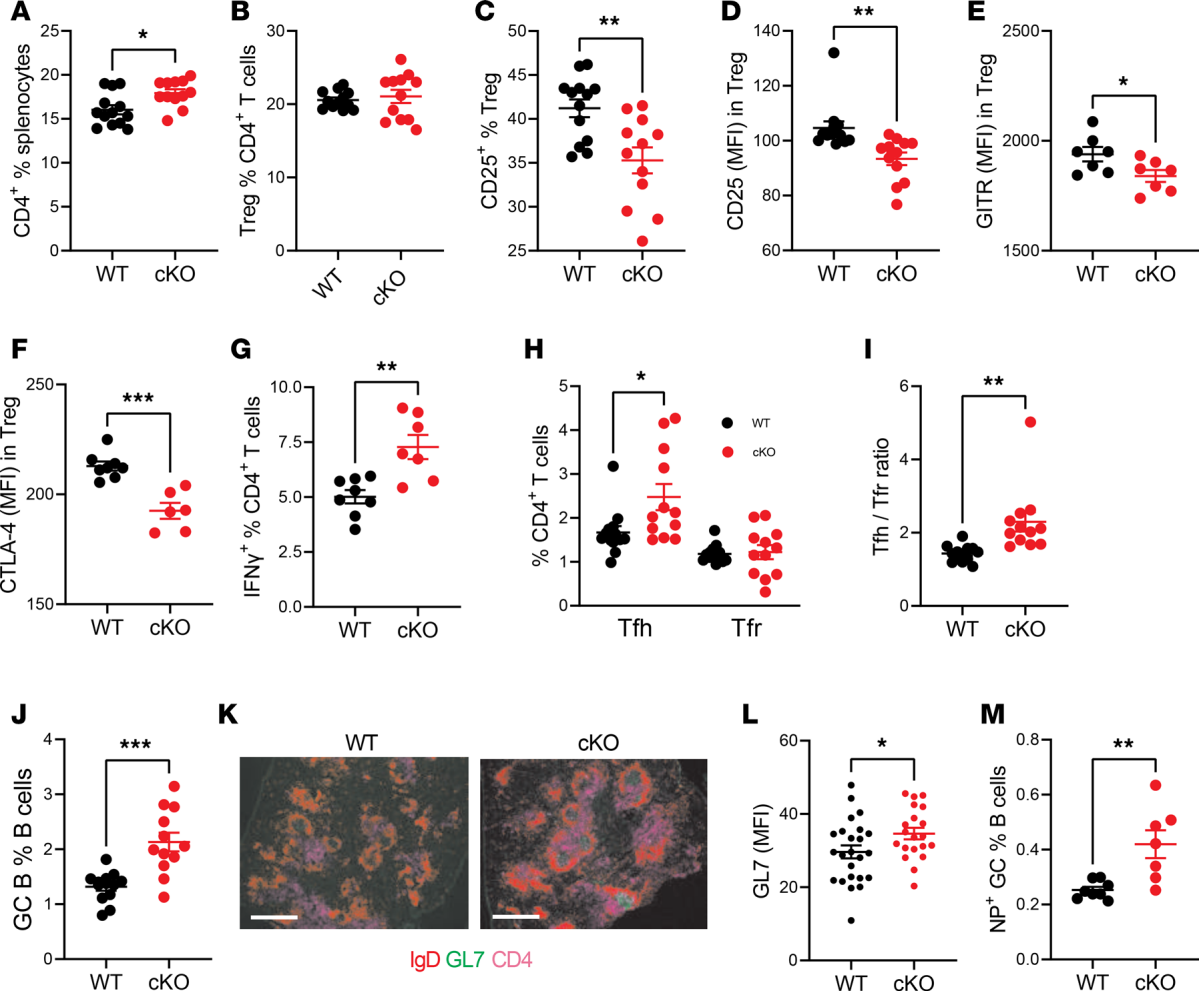
*Esrrg*-cKO mice show enhanced Tfh and GC responses to NP-KLH immunization. B6N.*Foxp3^YFP-Cre^* (WT) or B6N.*Esrrg*^fl/fl^ *Foxp3*^YFP-Cre^ (cKO) mice were immunized with NP-KLH/alum at week 0 and 2 and sacrificed at week 3. Percentage of splenic CD4^+^ T cells (**A**), FOXP3^+^ Tregs in CD4^+^ T cells (**B**), and CD25^+^ in Tregs (**C**). Expression (MFI) of CD25 (**D**), GITR (**E**), and CTLA-4 (**F**) on Tregs from immunized mice. Percentage of IFN-γ^+^ cells (**G**) and Tfh and Tfr subsets in the CD4^+^ T cells (**H**) in immunized mice. (**I**) Ratio of Tfh/Tfr in splenocytes. (**J**) Frequency of GC B cells in total B cells. (**K**) Representative GC B cell (GL7^+^, green) staining relative to non-GC B cells (IgD^+^, red) and CD4^+^ T cells (purple) in the spleen of immunized mice (original magnification, 4×; scale bar: 600 μm). (**L**) GL7 MFI was quantified in 3–5 GCs per mouse, *n* = 5 per group. (**M**) Percentage of NP^+^ GC B cells in B cells. Data combined from 3 independent experiments are shown as mean ± SEM, *n* = 4–12. Unpaired 2-tailed *t* tests, *P < 0.05, **P < 0.01, ***P < 0.001.

We tested the effect of *Esrrg* deficiency in Tregs in response to T cell–dependent (TD) immunization with nitrophenyl keyhole limpet hemocyanin (NP-KLH, Supplemental Figure 5A). Immunized *Esrrg-*cKO mice maintained the increased frequency of CD4^+^ T cells that was observed in unimmunized mice (Figure 3A). Although the frequency of Tregs was similar between the 2 groups of immunized mice, *Esrrg-*cKO Tregs expressed CD25, GITR, and CTLA-4 at a lower frequency and levels (Figure 3), which is consistent with the findings in unimmunized mice (Figure 2C). The frequencies of Tn and Tem cells and CD69^+^ T cells were similar between the 2 groups of immunized mice (Supplemental Figure 5, B–D), but we observed an increased frequency of IFN-γ^+^CD4^+^ T cells in the *Esrrg-*cKO mice (Figure 3G). Immunized *Esrrg-*cKO mice displayed an increased Tfh cell population and Tfh/Tfr ratio (Figure 3, H and I). Correspondingly, the frequency of GC B cells was increased in immunized *Esrrg-*cKO mice as measured by flow cytometry (Figure 3J) and histology (Figure 3, K and L). An increased frequency of nitrophenyl-specific (NP-specific) GC B cells was also found in *Esrrg-*cKO mice (Figure 3M), although the serum anti-NP IgG was comparable between groups (Supplemental Figure 5, E–G). These results suggest that *Esrrg* deficiency in Tregs enhanced Tfh and GC cellular responses to TD immunization.

### Esrrg deficiency in Tregs impairs Tfr cell differentiation.

**Figure 4:**
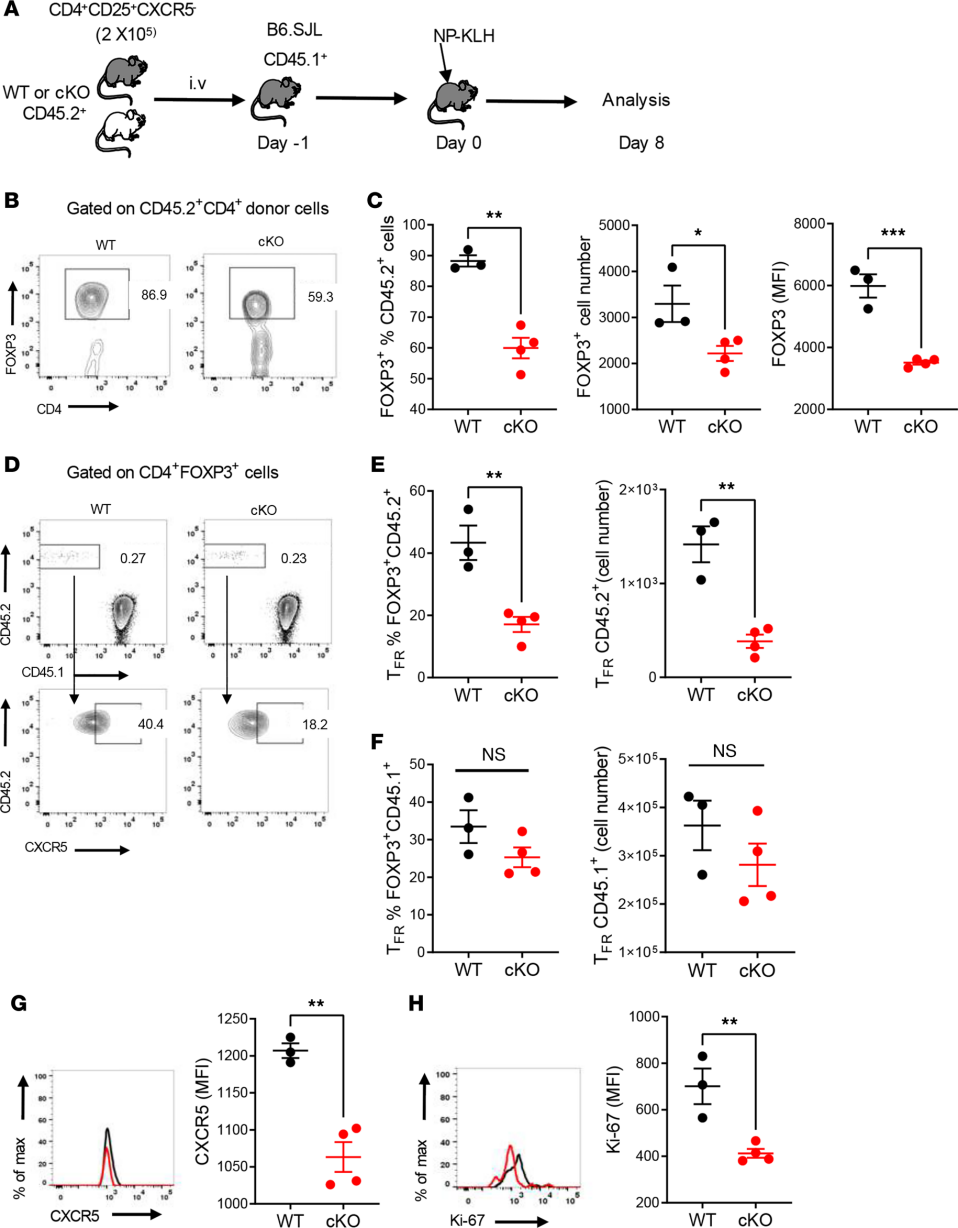
*Esrrg* deficiency in Tregs impairs Tfr cell differentiation. (**A**) Experimental design. Representative FACS plots of donor CD45.2^+^ Tregs (**B**) and their percentage, absolute number, and FOXP3 expression (**C**). (**D**) Representative FACS plots of donor CD45.2^+^ relative to recipient Tregs (Top) and donor Tfr cells (bottom). Percentage and absolute number of Tfr cells from donor (**E**) and recipient (**F**) origin. Representative FACS histograms and quantification of CXCR5 (**G**) and Ki-67 (**H**) expression in Tfr cells from donor origin. Data presented from 1 of 2 independent experiments with *n* = 4 in each are shown as mean ± SEM. Unpaired 2-tailed *t* tests, *P < 0.05, **P< 0.01, ***P < 0.001.

The increase in Tfh population and imbalance of Tfh/Tfr ratio observed at steady state and after immunization suggested that the differentiation of Tfr cells from *Esrrg-*cKO Treg precursors was impaired. To this end, CD45.2^+^CD4^+^CD25^+^CXCR5^–^ nonfollicular Tregs from either control or *Esrrg-*cKO mice were transferred into CD45.1^+^ B6.SJL mice followed by NP-KLH immunization (Figure 4A). The purity of the transferred nonfollicular Tregs was greater than 95% (Supplemental Figure 6, A and B). Eight days later, the frequency and number of CD4^+^FOXP3^+^ cells of *Esrrg-*cKO donor origin were dramatically reduced as compared with cells from control origin (Figure 4, B and C), and their FOXP3 expression was reduced (Figure 4C). These results indicate that *Esrrg* deficiency impaired Treg peripheral maintenance. In addition, the differentiation of CXCR5^–^ Tregs into CXCR5^+^ Tfr cells was reduced by half in the *Esrrg-*cKO donor cells (Figure 4, D and E). The frequency and number of CD45.1^+^ Tfr cells from recipient origin were similar between the 2 types of chimeric mice (Figure 4F), indicating that the impairment was intrinsic to the *Esrrg-*cKO Tregs. Furthermore, Tfr cells that differentiated from *Esrrg-*cKO Tregs expressed lower levels of CXCR5, while other markers, such as PD-1 and BCL6, were comparable between the groups (Figure 4G and Supplemental Figure 6C). Finally, Tfr cells that differentiated from *Esrrg-*cKO Tregs proliferated less than those differentiated from control Tregs (Figure 4H). These results suggest that *Esrrg* deficiency impaired the maintenance of Tregs and the formation of Tfr cells.

### Esrrg maintains mitochondrial metabolism in Tregs.

**Figure 5:**
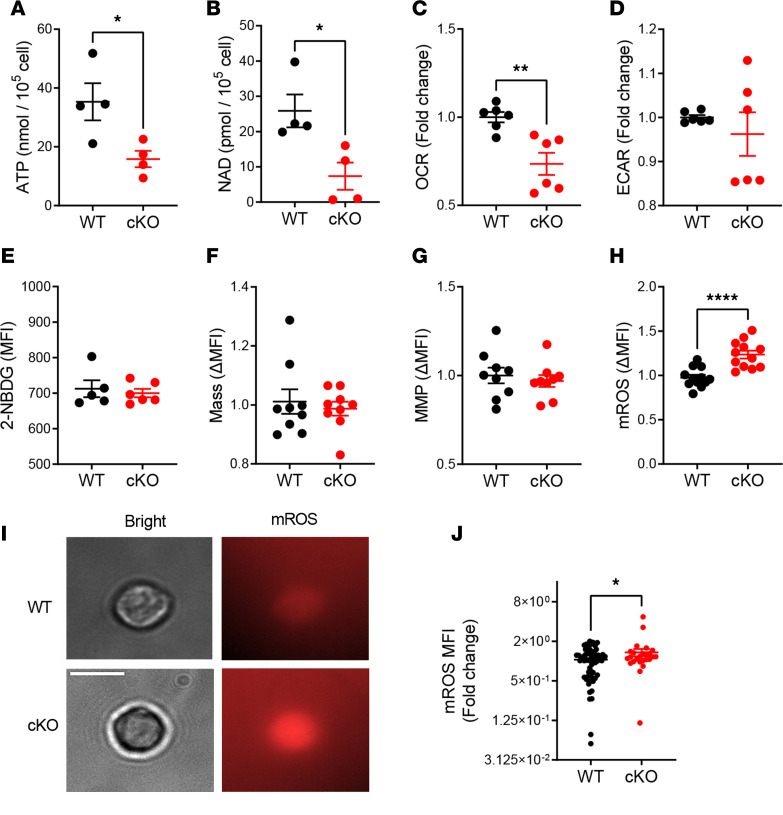
*Esrrg* maintains mitochondrial metabolism in Tregs. (**A** and **B**) ATP and NAD^+^ production by Tregs from 2- to 4-month-old B6N.*Foxp3*^YFP-Cre^ (WT) or B6N.*Esrrg*^fl/fl^ *Foxp3*^YFP-Cre^ (cKO) mice (*n* = 4). Basal OCR (**C**) and ECAR (**D**) in Tregs. Data were combined from 3 independent experiments with *n* = 2 each and expressed as fold change of the mean WT value for each experiment. (**E**) Glucose uptake in Tregs measured with 2-NBDG (*n* = 5–6). Mitochondrial mass (**F**), mitochondrial membrane potential (MMP, **G**), and mitochondrial ROS (mROS, **H**) in Tregs. Data were combined from 3 independent experiments with *n* = 3–4 each and expressed as fold change of the mean WT value for each experiment. Representative images of mROS staining in CD4^+^FOXP3^+^ cells (**I**) and quantification as MFI (**J**). Scale bar: 10 μm. Each symbol represents an individual Treg from WT (*n* = 62) and cKO (*n* = 25) mice. Data are shown as mean ± SEM. Unpaired 2-tailed *t* tests, *P < 0.05, **P < 0.01, ***P < 0.001.

*Esrrg* is essential for maintaining mitochondrial metabolism in several cell types (40, 48). Thus, we evaluated whether *Esrrg* deficiency altered the mitochondrial function of Tregs. *Esrrg-*cKO Tregs displayed a reduced production of ATP and NAD^+^ compared with control Tregs (Figure 5, A and B). Consistent with these results, *Esrrg-*cKO Tregs showed a reduced oxygen consumption rate (OCR) (Figure 5C), whereas their glycolysis measured by extracellular acidification rate (ECAR) was similar between groups (Figure 5D). Further investigation of glucose uptake measured by 2-NBDG [2-(*N*-(7-nitrobenz-2-oxa-1,3-diazol-4-yl)amino)-2-deoxyglucose] showed no differences between the groups (Figure 5E). Although mitochondrial mass and membrane potential were similar between groups (Figure 5, F and G), an elevated mitochondrial reactive oxygen species (mROS) production was observed in *Esrrg-*cKO Tregs (Figure 5H). This result was confirmed in sorted CD4^+^FOXP3^+^ Tregs stained with MitoSOX Red in which mROS levels were measured by microscopy (Figure 5, I and J). These results suggest that *Esrrg* is required for the optimal function of the mitochondrial electron transport chain in Tregs.

### Transcriptomic programming of Tregs by Esrrg.

**Figure 6:**
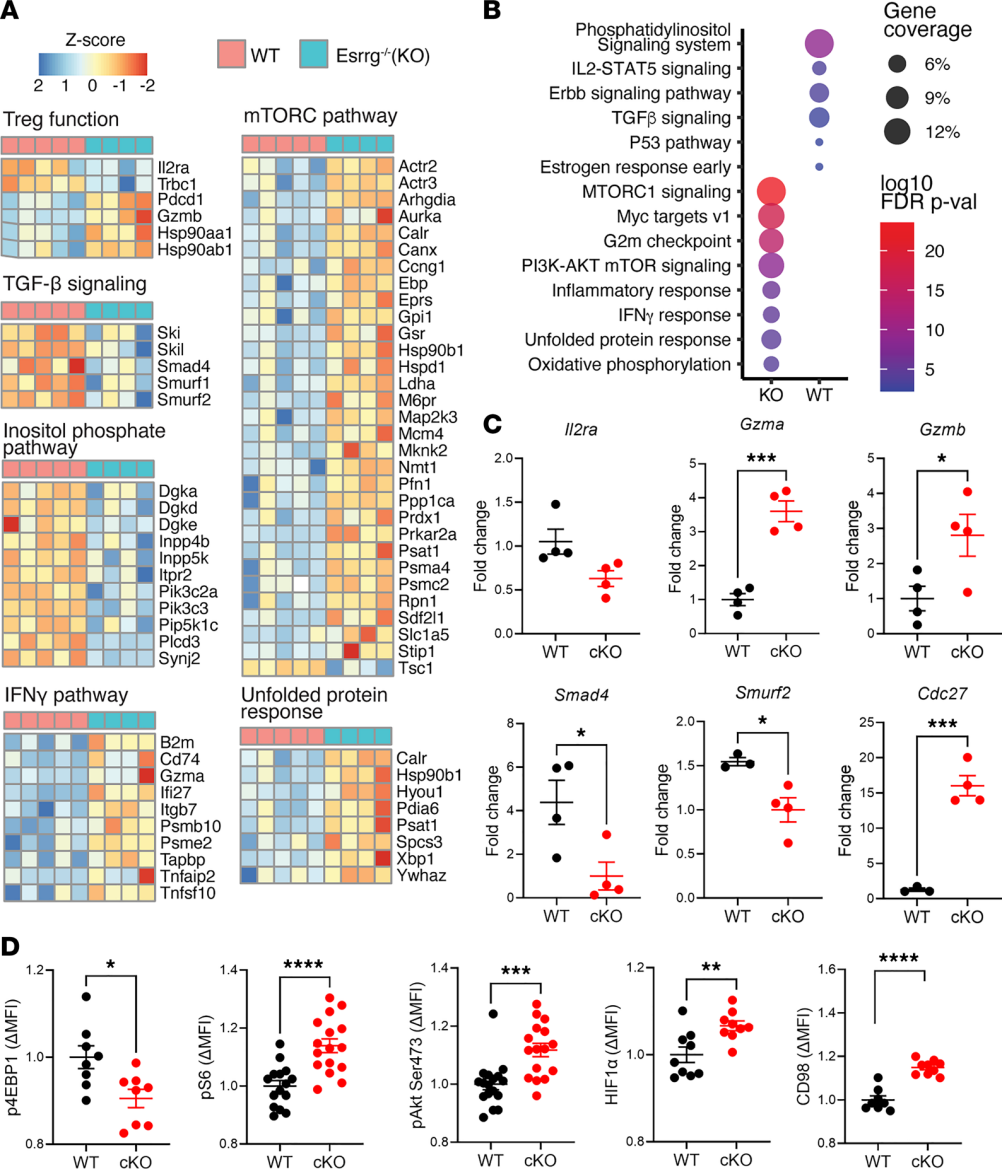
Transcriptomic programming of Tregs by *Esrrg*. (**A**) RNA-Seq analysis was conducted on sorted CD4^+^FOXP3^+^ Tregs from B6N.*FOXP3*^YFP-Cre^ (WT, *n* = 5) and B6N.*Esrrg*^fl/fl^ *FOXP3*^YFP-Cre^ (cKO, *n* = 4) mice. Heatmaps show a selection of differentially expressed genes in Treg-relevant pathways. (**B**) Hallmark analysis showing differentially enriched gene sets from WT and cKO mice. (**C**) qRT-PCR analysis on selected differentially expressed genes. Results are presented as fold change after being normalized to housekeeping gene *Hmbs* (*n* = 4). (**D**) pE4BP1, pS6, p-Akt Ser473, HIF1α, and CD98 protein expression in Tregs from WT and cKO mice. Data were combined from 2–3 independent experiments with *n* = 4–5 each and expressed as fold change of the mean WT value for each experiment. Data are shown as mean ± SEM. Unpaired 2-tailed *t* tests, *P < 0.05, **P < 0.01, ***P < 0.001, ****P < 0.0001.

To gain insights into how *Esrrg* regulates Treg activity, we performed RNA sequencing (RNA-Seq) of CD4^+^FOXP3^+^ T cells sorted from control and *Esrrg-*cKO mice at 2–3 months of age. Globally, 449 genes were upregulated in *Esrrg-*cKO Tregs while 447 genes were downregulated compared with control Tregs (*P* < 0.05, Supplemental Figure 7A). *Esrrg-*cKO Tregs showed decreased expression of genes associated with Treg function, such as *Il2ra* and *Trbc1* (Figure 6A). TGF-β signaling, inositol phosphate, and epidermal growth factor receptor (ERBB) pathways were significantly reduced in *Esrrg-*cKO Tregs (Figure 6, A and B). On the other hand, related metabolic pathways, such as mTOR signaling, MYC targets, and PI3K/AKT/mTOR, as well as IFN-γ signaling and inflammatory responses were dramatically enriched in *Esrrg-*cKO Tregs (Figure 6, A and B). Of note, expression of *Tsc1*, a negative regulator of mTOR activity that is essential to maintain the balance of Treg/Th17 cells (49), was decreased in *Esrrg-*cKO mice (Figure 6A). The unfolded protein response, which was enhanced in *Esrrg-*cKO Tregs (Figure 6, A and B), can be triggered by mitochondrial stress (50). It may further damage *Esrrg-*cKO Tregs by leaking out cytotoxic granules, inducing cleavage of cytoplasmic/nuclear substrates (51). The expression of OXPHOS transcripts was significantly augmented in *Esrrg*-cKO Tregs (Figure 6B and Supplemental Figure 6B), which may be a compensatory consequence of dysfunctional mitochondria evidenced with metabolic functional assays (Figure 5). A similar attempt to compensate for dysfunctional mitochondria may explain the strong upregulation of genes in the G2M pathway that was observed in *Esrrg*-cKO Tregs, although there is no evidence for their increased proliferation (Supplemental Figure 3C). The RNA-Seq results were validated by quantitative reverse-transcription PCR (qRT-PCR) on selected genes (Figure 6C). In addition, we used flow cytometry to validate the activation of the mTORC pathway, showing that pS6, phosphorylated AKT (p-AKT) Ser473, CD98, and HIF1α expression was elevated in the *Esrrg-*cKO Tregs compared with control Tregs, while the expression of pE4BP1 was reduced (Figure 6D). These results suggest a preferential activation of mTORC2 over mTORC1 in *Esrrg-*cKO Tregs, which is consistent with their enrichment in the PI3K/AKT/mTOR transcriptional pathway.

Interestingly, the SLE Gene Ontology pathway was enhanced in *Esrrg-*cKO Tregs (Supplemental Figure 6B). To better understand the relevance of *Esrrg*-cKO Tregs to SLE pathogenesis, we performed a gene set enrichment analysis (GSEA) to compare the ranked gene list of our RNA-Seq profiles with other publically available SLE-related CD4^+^ T cell gene sets (Supplemental Figure 6C). Several leading-edge gene sets (e.g., downregulated gene lists in the healthy vs. SLE CD4^+^ T cell comparisons) were significantly enriched in *Esrrg-*cKO group. These results support a functional link between the downregulation of *Esrrg* expression in Tregs and SLE.

### ESRRG contributes to the metabolism of human CD4^+^ T cells.

**Figure 7:**
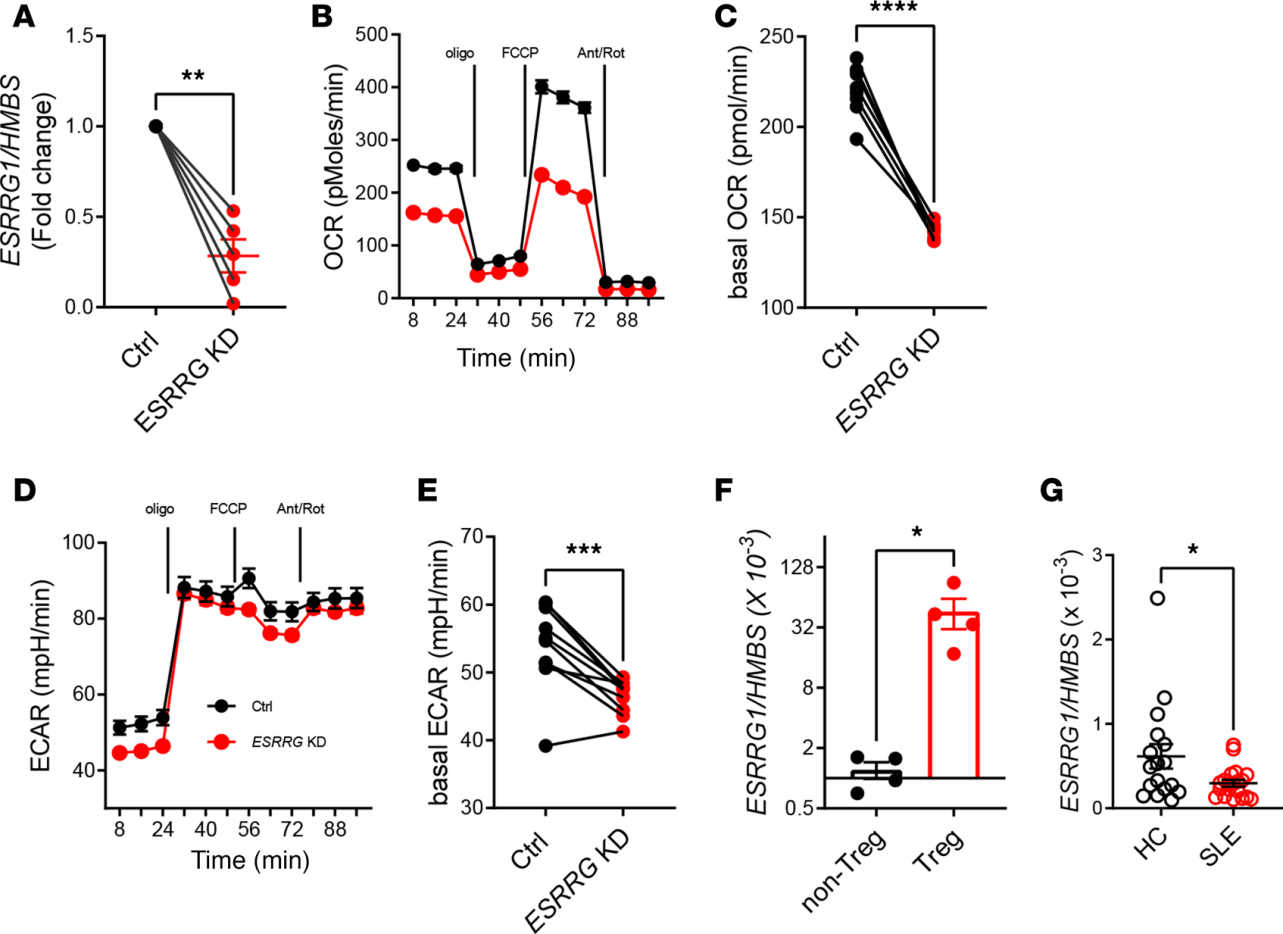
*ESRRG* expression in human CD4^+^ T cells. (**A**) *ESRRG* gene expression in control (Ctrl) and CRISPR-knockdown Jurkat T cells. (**B**–**E**) Mitochondrial stress test conducted on control and *ESRRG*-knockdown Jurkat T cells. Representative OCR (**B**, *n* = 2) and basal OCR (**C**). Representative ECAR (**D**, *n* = 2) and basal ECAR (**E**). Results are shown relative to control cells for each experiment (*n* = 10 combined from 4 independent experiments). (**F**) *ESRRG* expression in Treg and non-Treg CD4^+^ T cells from healthy donors. (**G**) *ESRRG* expression in total CD4^+^ T cells from healthy controls (HC, *n* = 17) and SLE patients (*n* = 20). Data are shown as mean ± SEM. Two-tailed paired *t* tests (**A**, **C**, and **E**) and unpaired *t* tests (**F** and **G**), **P < 0.01, ***P < 0.001.

To explore the role of *ESRRG* in human T cells, we first targeted *ESRRG* in the human Jurkat T cell line using CRISPR/Cas9 technology. CRISPR-mediated deletion lowered *ESRRG* expression to 20% of the control group (Figure 7A) and reduced both mitochondrial oxidation and glycolysis (Figure 7, B–E). Our attempts to edit *ESRRG* in primary human Treg or total CD4^+^ cells were unsuccessful because the stimulation/electroporation eliminated *ESRRG* expression, which was very low to start with. This finding is consistent with results obtained with murine CD4^+^ T cells in which we showed that activation reduced *Esrrg* expression (37). Nonetheless, these results support the notion that *ESRRG* contributes to human CD4^+^ T cell metabolism. Next, we evaluated *ESRRG* expression in human Tregs and showed that it was higher than in the corresponding non-Treg CD4^+^ T cells (Figure 7F). Finally, we have previously shown that *Esrrg* expression is lower in the CD4^+^ T cells from the NZM2410 lupus-prone mice and the B6.*Sle1c2* congenic mice carrying the NZM2410 *Esrrg* allele (37). Here, we show that CD4^+^ T cells from SLE patients also expressed a lower level of *ESRRG* as compared with healthy controls (Figure 7G). Overall, we found a similar association between low *ESRRG* expression in T cells and lupus susceptibility in mice and humans, and our results suggest that *ESRRG* may contribute to human T cell metabolism.

## Discussion

A better understanding of the regulation of Treg survival and function has provided insights into therapeutic strategies for SLE. It has been proposed that restoration of self-tolerance by increasing the number or function of Tregs could be beneficial in patients with SLE (52). In support of this hypothesis, a treatment with low-dose IL-2 boosted Treg numbers and alleviated disease activity in patients with SLE (53, 54). Further, the combination of low-dose IL-2 with rapamycin normalized the balance of Th17/Tregs and reduced disease activity in refractory SLE patients (55). Rapamycin is an inhibitor of mTORC1, which prevents FOXP3 expression and impairs Treg function (22). Further, the elevated level of IL-21 found in SLE patients impairs Tregs by activating mTORC1 and mTORC2 (56). Taken together, these results support targeting Treg dysfunction as a therapeutic strategy to treat SLE. Tregs are acutely dependent on cellular and mitochondria metabolism as shown by the loss of suppressive activity in Treg-specific deficiency of mTOR, *Lkb1*, *Uqcrsf1*, and *Tfam* (28, 29, 31, 32, 57). Consistent with these findings, CD4^+^ T cells from mice carrying the lupus susceptibility locus *Sle1c2* express a low level of *Esrrg* and present mitochondrial defects (37). Here we show that the *Esrrg*-cKO Tregs were defective and presented mitochondrial defects with increased ROS production coupled with decreased respiration as well as reduced ATP and NAD^+^ production. We also showed that *ESRRG* expression was lower in the CD4^+^ T cells of SLE patients as compared with healthy controls. Further, *ESRRG* was expressed at a higher level in human Tregs than in the non-Treg CD4^+^ T cell fraction. Finally, knocking down the expression of *ESRRG* in a human T cell altered its metabolism, supporting the hypothesis that *ESRRG* may also regulate human T cell function through T cell metabolism.

*Esrrg* deficiency did not alter glycolysis in murine Tregs, further supporting that the impaired Treg functions resulted from a defective mitochondrial metabolism rather than increased glycolysis. *Esrrg* deficiency in Tregs increased mTORC2, which is likely to play a major role in their impaired function (21). A causal link between impaired mitochondrial function and mTOR activation has been demonstrated in Leigh syndrome (58), and it is most likely that it also applies to *Esrrg*-cKO Tregs. Furthermore, mTORC2 and the PI3K/AKT/mTOR pathway are activated by mROS (59, 60). These results suggest that *Esrrg* deficiency impairs mitochondrial function, increasing mROS production, which overactivates mTOR and impairs Treg functions. It has been shown that mTOR inhibition ameliorates mitochondrial functions in SLE T cells (61). It is therefore possible that *Esrrg* regulates a crosstalk between mitochondria and mTOR in Tregs. Further studies will be necessary to establish a causal link between low *ESRRG* expression and Treg dysfunction in SLE patients.

There is an expanded CD8^+^CD38^hi^ T cell population in a group of SLE patients with recurring infections that are characterized by decreased granzyme-dependent cytotoxicity and an impaired NAD^+^ production in a NAD/SIRTUIN1/EZH2 axis (62). However, CD38, EZH2, and related transcription factors TBET, RUNX3, and EOMES were not affected in *Esrrg-*cKO Tregs, suggesting that in our model, NAD^+^ and granzyme production are independent from the SIRTUIN1/EZH2 axis. *Esrrg* deficiency in Tregs increased the expression of malate dehydrogenase 2 (*Mdh2*) and succinyl-CoA ligase subunit 1 (*Suclg1*). MDH2 plays a major role in the malate-aspartate shuttle, providing NAD^+^ to the TCA cycle to convert into NADH. This may contribute to decreased NAD^+^ in *Esrrg-*cKO Tregs. SUCLG1 catalyzes the conversion of succinyl-CoA to succinate. Succinate accumulates with mitochondrial complex III defects and decreased OXPHOS, which impairs Tregs (31). These findings may indicate a direct effect of *Esrrg* on the TCA cycle.

*Esrrg* deficiency also resulted in the downregulation of major pathways involved in Treg functions. PI3K activity is crucial for the maintenance of effective immunity and Treg suppressive function in both mouse models and human cells (63, 64). Mice lacking PI3K in Tregs display spontaneous peripheral nerve inflammation in the experimental autoimmune encephalomyelitis model (65). TGF-β is essential for pTregs to differentiate from naive CD4^+^ T cells and to maintain Treg homeostasis (66). Interestingly, during T cell activation, TGF-β signaling regulates kinase networks and phosphatidylinositol metabolism through SMAD3 (67). *Esrrg-*cKO Tregs present reduced transcripts in both of these pathways, suggesting that *Esrrg* may regulate phosphatidylinositol metabolism via TGF-β signaling. Finally, the EGFR in the ERBB pathway plays a crucial role in Treg suppressive function (68) by maintaining *Foxp3* expression (69). Overall, the genetic pathways controlled by *Esrrg* as well as the phenotypes of *Esrrg-*cKO Tregs suggest that this gene regulates the maintenance and fitness of pTregs rather than tTreg differentiation.

Tfr cells differentiate from naive tTregs, migrate to the GC, and inhibit Tfh and GC B cells. The importance of Tfr cells in immune tolerance was demonstrated in Bcl6^fl/fl^ Foxp3^Cre^ Tfr -deficient mice that develop excessive Tfh cells and spontaneous autoimmune disease (70). We observed an increased frequency of Tfh cells and a dysregulated Tfr/Tfh ratio in the *Esrrg-*cKO mice at steady state and upon TD immunization. Consistent with this finding, the differentiation and the proliferation of Tfr cells were impaired by *Esrrg-*cKO Tregs, and *Esrrg-*cKO Tfr cells expressed lower levels of CXCR5, which impairs their migration to the GC. FOXP3 expression in Tfr cells is essential for modifying the Tfh program and enforcing suppression. Moreover, a reduction of FOXP3 expression, as we observed in *Esrrg*-cKO Tfr cells, promotes the formation of ex-Tfr cells with a potential pathogenic activity (71). A low expression of *Esrrg* could therefore contribute to lupus phenotypes by affecting both Treg and Tfr cells, in a relative contribution that needs to be further evaluated.

*Esrrg* plays an important role in supporting mitochondrial metabolic fitness in various cellular processes (38). Another orphan nuclear receptor, *Esrra*, promotes T cell effector functions through glucose metabolism (72). Here, we report a potentially novel role for *Esrrg* in the immune system by controlling the metabolic fitness and function of Tregs through their mitochondria. It is intriguing that these 2 related transcription factors regulate T cell responses in opposite directions, with *Esrra* promoting and *Esrrg* preventing inflammation, through opposite metabolic pathways. This suggests that a better understanding of these interacting processes may uncover novel metabolic regulatory checkpoints of T cell regulation.

## Methods

### Mice.

B6.SJL-*Ptprc*^a^*Pepc*^b^/BoyJ (B6.SJL, 002014), B6.129(Cg)-*Foxp3*^tm4(YFP/icre)Ayr^/J (B6J.*Foxp3*^YFP-Cre^, 016959), C57BL/6NJ (B6N, 005364), and B6.129S7-*Rag1*^tm1Mom^ (B6.*Rag*^–/–^, 002216) were originally bought from the Jackson Laboratory on a B6J genetic background with the indicated strain numbers. B6N.*Esrrg*^fl/fl^ mice (Supplemental Figure 1) were generated by genOway and bred with B6J.*Foxp3*^YFP-Cre^ to generate *Esrrg*^fl/fl^ *N*. *Foxp3^YFP-Cre^* (cKO reporter mice). B6N mice were bred with B6J.*Foxp3*^YFP-Cre^ to generate B6N.*Foxp3*^YFP-Cre^ (WT reporter mice). All mice were maintained on the NAD(P) transhydrogenase (*Nnt*) WT allele (N). Nnt is a mitochondrial enzyme involved in NAD synthesis. The *Nnt*-null allele in B6J may interfere with some of the *Esrrg-*cKO phenotypes, which would complicate data interpretation. All experiments were conducted according to protocols approved by the University of Florida (UF) Institutional Animal Care and Use Committee. Only female mice were used in this study. The initial characterization of male cKO mice showed variable phenotypes with less consistent differences with WT, as compared with female mice. Unless specified in the article, control refers to B6N.*Foxp3*^YFP-Cre^ mice, and *Esrrg-*cKO refers to B6N.*Esrrg*^fl/fl^.*Foxp3*^YFP-Cre^ mice.

### Flow cytometry.

Single-cell suspensions were prepared using standard procedures from spleen, thymus, and mLN. After RBC lysis, cells were stained in FACS staining buffer (2.5% FBS, 0.05% sodium azide in PBS). Fluorochrome-conjugated Abs are as follows: B220 (clone RA3-6B2), BCL6 (clone K112-91), CD45.1 (clone A-20), CD62L (clone MEL-14), CD95 (clone Jo2), CXCR5 (clone 2G8), IgD^b^ (clone 217-170), and Ly6C (clone AL-21) were purchased from BD Biosciences. CD4 (clone RM4-5), CD8a (clone 53-6.7), CD45.2 (clone 104), CD103 (clone 2e7), CD122 (clone TM-B1), CD132 (clone TUGm2), CD138 (clone 281-2), CTLA-4 (clone UC10-4B9), IL-10 (clone JES5-16E3), IL-17a (clone TC11-18h10.1), and IFN-γ (clone XMG1.2) were purchased from BioLegend. CD4 (clone GK1.5), CD44 (clone IM7), CD25 (clone PC61.5), CD69 (clone H1.2F3), CD98 (clone RL388), CD127 (clone 199), CD279 (clone RMP1-30), CD357 (clone DTA-1), FOXP3 (clone FJK-16S), GL-7 (clone GL-7), GITR (clone DTA-1), HELIOS (clone 22F6), Ki-67 (clone SolA15), p-AKT (clone SDRNR), and PD-1 (clone RMP1-30) were purchased from eBioscience, Thermo Fisher Scientific. Nrp1 (clone ACY10212121) and Hif1-a (clone 241812) were purchased from R&D Systems, Bio-Techne. PE4-BP1 (clone 236B4) and P-S6 (clone D57.2.2E) were purchased from Cell Signaling Technology. Follicular T cells were stained in a 3-step process using purified CXCR5 (2G8, BD Biosciences) followed by biotinylated anti-rat IgG (Jackson ImmunoResearch) and PerCP5.5-labeled streptavidin in FACS staining buffer on ice. The eBioscience Fixation/Permeabilization kit (Thermo Fisher Scientific) was used for intracellular staining. For cytokines, cells were seeded in 96-well plates with 200 μL RPMI containing Leukocyte Activation Cocktail (BD Biosciences) for 4 hours. Then cells were washed and stained with surface markers and followed with Fixation/Permeabilization and intracellular staining. Cell samples were acquired on LSRFortessa (BD Biosciences) and analyzed with FlowJo (Tree Star).

### Histology and immunofluorescence staining.

Spleen, colon, and lung sections were stained with H&E. Images were digitized and quantitation of the number of GC and immune foci was performed by applying the same gate of approximately one-half of each section to each sample. Immunofluorescence staining of GCs was performed on frozen spleen sections with anti-GL7-FITC (GL7; 1:25), anti-IgD^b^-PE (217-170, 1:50), and anti-CD4-APC (RM4-5, 1:100), all from BD Biosciences, as previously described (13).

### Autoantibody detection.

For ANA detection, serum was diluted 1:40 and used for indirect staining of Hep-2 slides (Bio-Rad) with Alexa Fluor 488–conjugated goat anti-mouse IgG (BD Biosciences). Slides were fixed with Fluoromount (MilliporeSigma), and fluorescence intensity was quantified with the ImageJ software (NIH). Anti-dsDNA IgG was detected as previously described by ELISA (73) with serum diluted 1:100.

### In vitro and in vivo Treg suppression assay.

Tregs from control and *Esrrg*-cKO splenocytes were isolated, either purified using Treg isolation kit II (Miltenyi Biotec) with the AutoMACS Pro (Miltenyi Biotec) or sorted as FOXP3-YFP^+^ with the S3e Cell Sorter (Bio-Rad). CD45.1^+^CD4^+^CD25^–^ Teff cells from B6.SJL mice were labeled with CellTrace Violet (CTV) (Life Technologies, Thermo Fisher Scientific). Dendritic cells (DCs) were isolated from B6N mice by positive selection with anti-CD11c isolation kit (Miltenyi Biotec). CD45.2^+^CD4^+^CD25^+^ Tregs were incubated with Teff cells (6.6 *×* 10^4^) at a 1:1 to 1:4 ratio in the presence of DCs (1 *×* 10^4^) and soluble anti-CD3e antibody (1 μg/mL, BD Biosciences 145-2C11) for 3 days (74). The proliferation of CD45.1^+^ Teff cells was determined by the dilution of CTV, and the proliferation index was calculated with the FlowJo software.

For the experimental colitis model, *B6.Rag^–/–^* mice were injected i.v. with sorted CD4^+^FOXP3^–^ Teff cells (0.4 *×* 10^6^) either alone or with control or *Esrrg-*cKO Tregs (0.1 *×* 10^6^). Mice were weighed and examined weekly and euthanized at week 13. Splenocytes were analyzed with flow cytometry, and colons were fixed and stained with H&E or anti-CD45 (1:25 dilution; BD Pharmingen 30F11). Infiltrating lymphocytes in colon were quantified with Imagescope.

### Treg differentiation and RNA silencing of Esrrg.

CD4^+^CD25^–^ T cells from control or *Esrrg*-cKO mice were cultured under the Treg polarizing conditions. Briefly, 1 *×* 10^6^ T cells were incubated with plate-bound anti-CD3e and soluble 1 μg/mL anti-CD28 antibody (37.51, BD Biosciences) in addition to 3 ng/mL TGF-β (PeproTech), 50 ng/mL IL-2 (R&D Systems, Bio-Techne), 10 μg/mL anti–IFN-γ (XMG1.2, BioXcell), and anti-IL-4 (11B11, BioXcell) antibodies in complete RPMI media for 4 days (75). *Esrrg* RNA silencing was conducted with FANA oligonucleotides (Aumbiotech). Briefly, CD4^+^CD25^–^ T cells from B6N mice were stimulated with anti-CD3e and CD28, together with *Esrrg* or scrambled FANA oligonucleotides at the concentration of 10 nM for 2 days, then switched to Treg polarizing conditions for another 3 days, followed by flow cytometry analysis.

### Immunization and antibody measurements.

WT or *Esrrg*-cKO mice, 2–3 months old, were immunized with 100 μg NP_23_-KLH in alum (1:1) at week 0 and boosted at week 2. Serum samples were collected at weeks –1, 1, and 3. Serum NP-specific IgG1 were determined as previously described (13) by ELISA using plates coated with NP_4_-BSA or NP_25_-BSA (Biosearch Technologies), followed by incubation with 1:500 diluted serum samples and developed with alkaline phosphatase–conjugated goat anti-mouse IgG1. All samples were run in duplicate.

### Tfr differentiation in vivo.

B6.SJL (CD45.1^+^) mice were adoptively transferred with 2 *×* 10^5^ CD4^+^CD25^+^CXCR5^–^ cells sorted from either control or *Esrrg-*cKO Treg CD45.2^+^ mice, followed by immunization with NP-KLH in alum. Splenocytes were analyzed by flow cytometry on day 8 for Tfr cell differentiation as described (76).

### Metabolic measurements.

OCR and ECAR were measured as previously described (75) with XF96 Extracellular Flux Analyzer under mitochondrial stress test conditions (Seahorse, Agilent). The concentration of oligomycin, carbonyl cyanide-4-phenylhydrazone, rotenone, and antimycin A (all from MilliporeSigma) were set as 1 μM, 1.25 μM, 1 μM, and 1 μM, respectively. NAD^+^ was measured in Tregs sorted from 2-month-old control and *Esrrg-*cKO mice using the NAD/NADH Quantitation Kit (MilliporeSigma, MAK037). Total amount of ATP was measured in these cells with the ATP Determination Kit (Thermo Fisher Scientific). Luminescence was detected with a Lumat LB 9507 luminometer (Berthold Technologies).

### Mitochondrial ROS, mass, and membrane potential.

Mitochondrial ROS was measured by MitoSOX Red. Mass and membrane potential were tested by MitoTracker Red CMXRos and MitoTracker Deep Red (all from Thermo Fisher Scientific). The assay was performed on LSRFortessa and analyzed with FlowJo. For mitochondrial ROS imaging, sorted CD4^+^FOXP3^–^YFP^+^ Tregs were stained with MitoSOX Red and assessed on a fluorescence microscope. MFI of each single Treg was calculated with ImageJ.

### RNA-Seq analysis.

Total RNA was extracted from sorted control and *Esrrg-*cKO CD4^+^FOXP3^YFP+^ Tregs using the RNeasy Kit (QIAGEN). RNA-Seq libraries were prepared with the TruSeq Stranded RNA Sample Preparation Kit (Illumina). The quality was validated with the Agilent Bioanalyzer 2100. The amplified libraries were size-selected and quantified with the PicoGreen assay (Life Technologies, Thermo Fisher Scientific). RNA-Seq reads were mapped to the *Mus musculus* genome mm10 using STAR aligner (v2.7) (77). FeatureCounts (v2.0) was used to calculate gene counts for each sample (78). Significantly altered genes (FDR *P* < 0.05) were identified by DESeq2 (79). Significant overlaps with other Hallmark gene sets in MSigDB were computed on the MSigDB website (www.gsea-msigdb.org/gsea/msigdb/annotate.jsp). Regularized log transformation of count data was performed for heatmap plotting. The GSEA was performed in javaGSEA (v3.0) using rank lists based on Wald’s statistics, which are generated from the DESeq2 analysis (80). The gene sets used for the analyses were retrieved from MSigDB v7.1 (81, 82).

### qRT-PCR.

The *Esrrg* TaqMan probe (Thermo Fisher Scientific, Mm01318905_m1) was used for mouse cells. SYBR Green (Bio-Rad) was used to quantify gene expression for RNA-Seq validation on the Bio-Rad CFX connect system with primer sequences shown in Supplemental Table 1. Expression was calculated using the method with difference in 2^ΔCt^ values normalized to the housekeeping gene *Hmbs* or *Ppia* against the gene of interest. *ESRRG* expression was measured in CD4*^+^* T cells isolated from the peripheral blood of SLE patients and healthy controls (83) with the primers shown in Supplemental Table 1.

### Human ESRRG expression and CRISPR/Cas9 editing.

For CRISPR/Cas9 editing, Jurkat T cells (1 *×* 10^6^) (Thermo Fisher Scientific) were electroporated with the Amaxa P3 Primary Cell Kit and 4D-Nucleofecter selected program EH100 (Lonza). Guide RNAs were prepared with 3 chemically synthesized pooled RNAs (Synthego) targeting exon 3 of *ESRRG* (5′-CUGAAGAGCCACCAGGGCUG-3′, 5′-GUUGACGCUGUCCGUCAGGG-3′, and 5′-CUUGAUGAAGGACGAACAGC-3′). Ribonucleoprotein complexes were mixed in a sterile microcentrifuge tube with 120 pmol sgRNA and 20 pmol Cas9 (6:1) in nucleofector P3 primary solution. After electroporation, cells were cultured in complete RPMI for 3 days, when *ESRRG* gene expression relative to housekeeping gene *HMBS* expression (primer sequences shown in Supplemental Table 1) was measured and a mitochondrial stress Seahorse assay was performed. *ESRRG* gene expression was also measured in total CD4^+^ T cells from the cohort of SLE patients and healthy controls previously recruited (83), as well as in Treg versus non-Treg CD4^+^ T cells. For the latter, peripheral blood was obtained from healthy donors. PBMCs were isolated with gradient centrifugation using Ficoll-Paque (GE Healthcare), and CD4^+^CD25^+^CD127^dim^ Tregs were isolated with regulatory T cell isolation kit (Miltenyi Biotec).

### Data and code availability.

The accession number of the RNA-Seq data from control and *Esrrg*-cKO Tregs files reported in this paper is Gene Expression Omnibus GSE150187 (https://www.ncbi.nlm.nih.gov/geo/).

### Statistics.

Unless specified, differences between groups were evaluated by unpaired 2-tailed *t* tests. The results are expressed as means ± SEM. The statistical analysis was performed with the GraphPad Prism 8.0 software. The levels of statistical significance were set at *P < 0.05, **P < 0.01, ***P < 0.001, and ****P < 0.001.

### Study approval.

All mouse experiments were conducted according to protocols approved by the UF Institutional Animal Care and Use Committee. CD4^+^ T cells from SLE patients and healthy controls were obtained from the UF Lupus Clinic (UF IRB approval 201300225).

## Author contributions

WL and LM designed the experiments and analyzed results. WL, SCC, ASE, AAT, NK, and JB conducted cellular experiments. MG performed the RNA-Seq analysis. WL, WY, LP, and YPP conducted CRISPR experiments. JB and ASE performed tissue processing and histology. SCC, XT, and TMB contributed to experimental design and data interpretation. WL and LM wrote the manuscript.

## Supplementary Material

Supplemental data

## Version 1 06/22/2021

In-Press Preview

## Version 2 07/22/2021

Electronic publication
