# Supplementary material for: Lupus susceptibility gene Esrrg modulates regulatory T cells through mitochondrial metabolism
Source: JCI Insight. 2021 Jul 22;6(14):e143540. doi: 10.1172/jci.insight.143540 (PMC8410062; doi:10.1172/jci.insight.143540)
Supplement: Supplemental data [file jciinsight-6-143540-s043.pdf]

Supplementary Materials

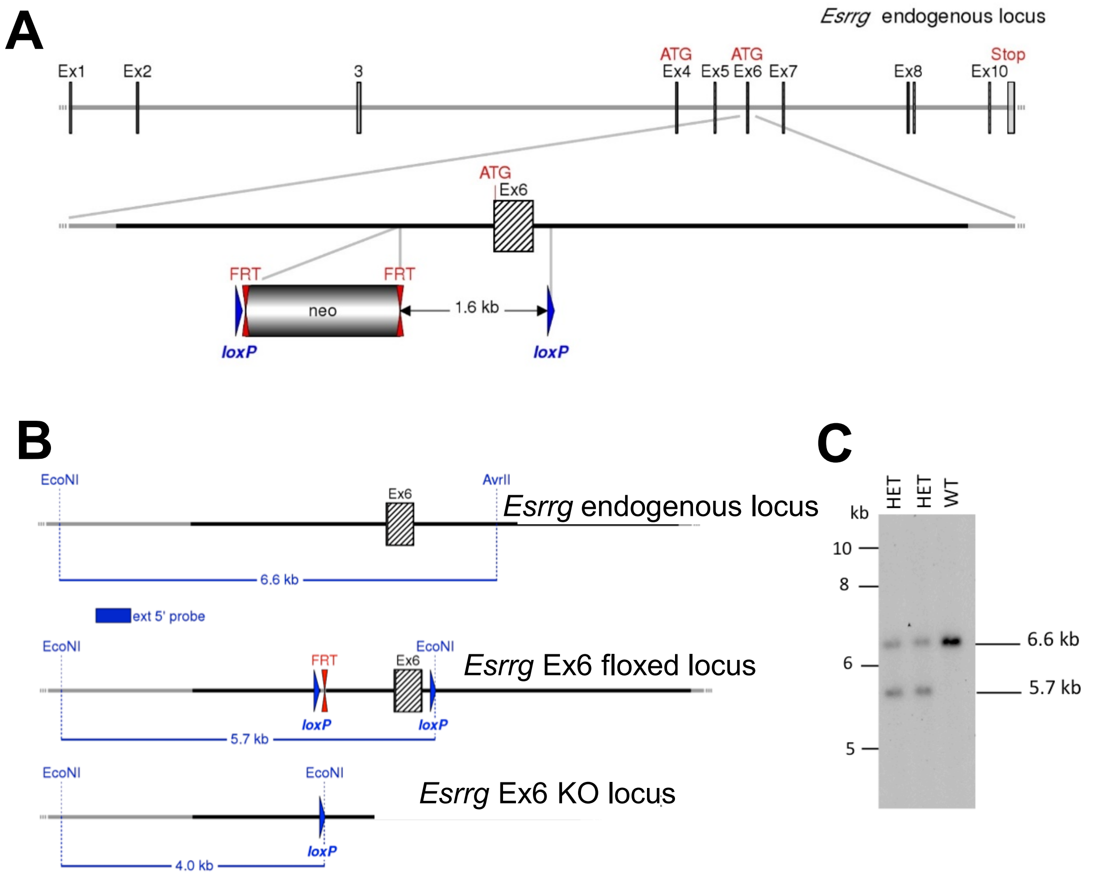

Figure S1. ***Esrrg* deletion construct.** (**A**) Targeting strategy to delete exon 6. Diagram is not depicted to scale. Hatched rectangles represent coding sequences, grey rectangles indicate non-coding exon portions, solid lines represent chromosome sequences. The neomycin positive-selection cassette is indicated. loxP sites are represented by blue triangles and FRT sites by double red triangles. The initiation (ATG) and Stop (Stop) codons are indicated. The size of the flanked sequence to be deleted is specified. (**B**) *Esrrg* wild-type endogenous, floxed exon 6 and deleted exon 6 alleles. The location of the Southern blot probe, restriction sites and corresponding fragment sizes are indicated. (**C**) Representative results of Southern blot analysis. The genomic DNA of the heterozygous conditional Knock-out tested animals (HET) was compared to wild-type DNA (WT). The AvrII - EcoNI digested DNAs were blotted on nylon membrane and hybridised with an external 5' probe. The construct and deletion were also verified by PCR.

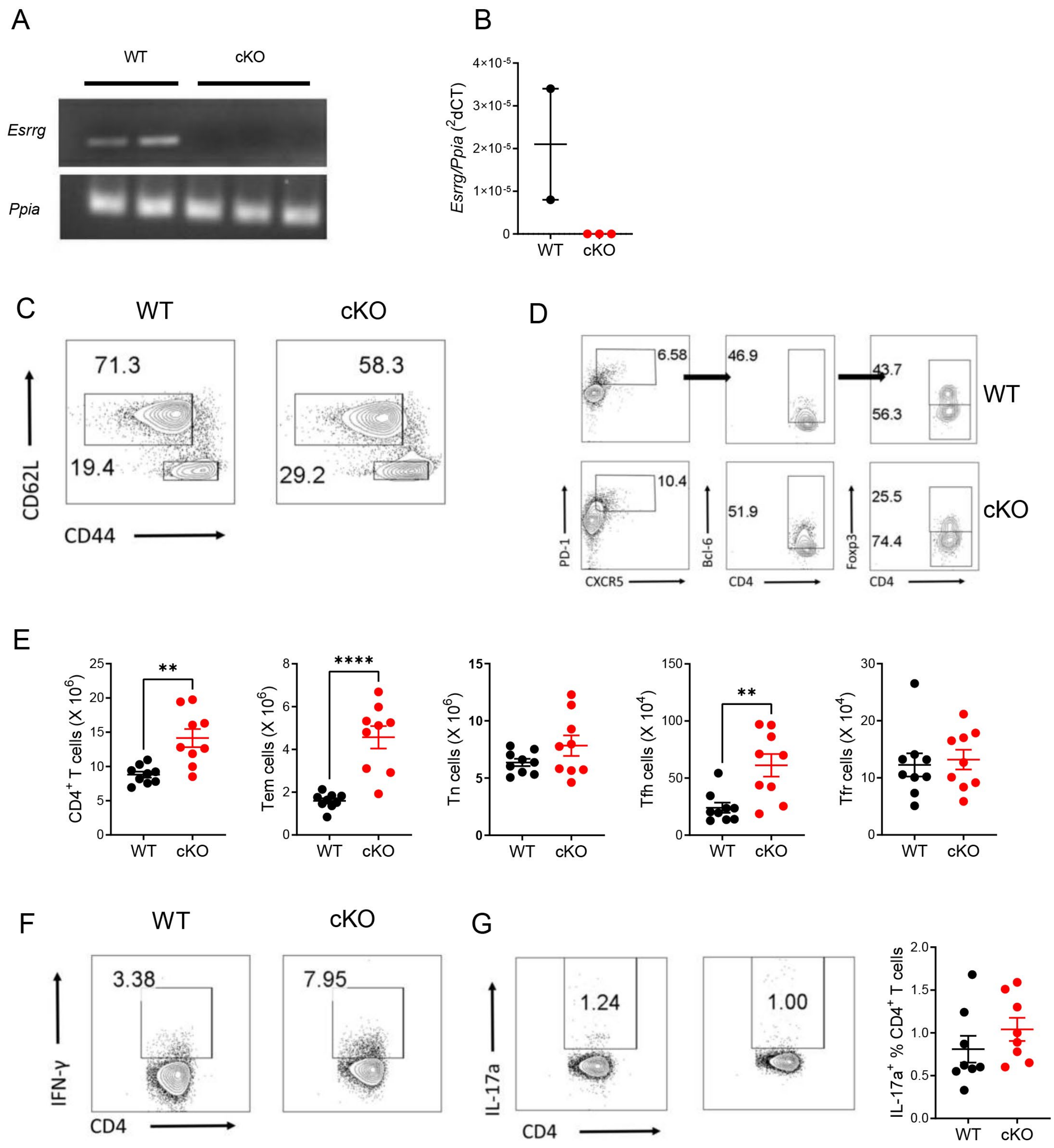

Figure S2. *Esrrg* deficiency in Treg cells leads to CD4⁺ T cell activation and autoimmunity in aged mice. (**A**, **B**) *Esrrg* mRNA expression in CD4⁺Foxp3⁺ Treg cells from B6N.*Foxp3*$^{\text{YFP-Cre}}$ (WT) and B6N.*Esrrg*$^{f/f}$ *Foxp3*$^{\text{YFP-Cre}}$ (cKO) mice with conventional (**A**) PCR and qRT-PCR (**B**). *Ppia* was used as control. Representative $CD4^+$-gated FACS plots showing the $CD44^-CD62L^+$ Tn and $CD44^+CD62L^-$Tem subsets (**C**), as well as $Bcl6^+PD1^+CXCR5^+FOXP3^-$ Tfh and $Bcl6^+PD1^+CXCR5^+FOXP3^+$ Tfr subsets (**D**). (**E**) Absolute splenic cell numbers of $CD4^+$ T, Tem, Tn, Tfh and Tfr cells. (**F**) Representative $CD4^+$-gated FACS plots showing IFN-γ^+ cells. (**G**) Representative $CD4^+$-gated FACS plots showing IL-17a$^+$ cells and their quantification on the right. Data are shown as mean ± SEM. Unpaired t-tests, ** $P < 0.01$, **** $P < 0.0001$.

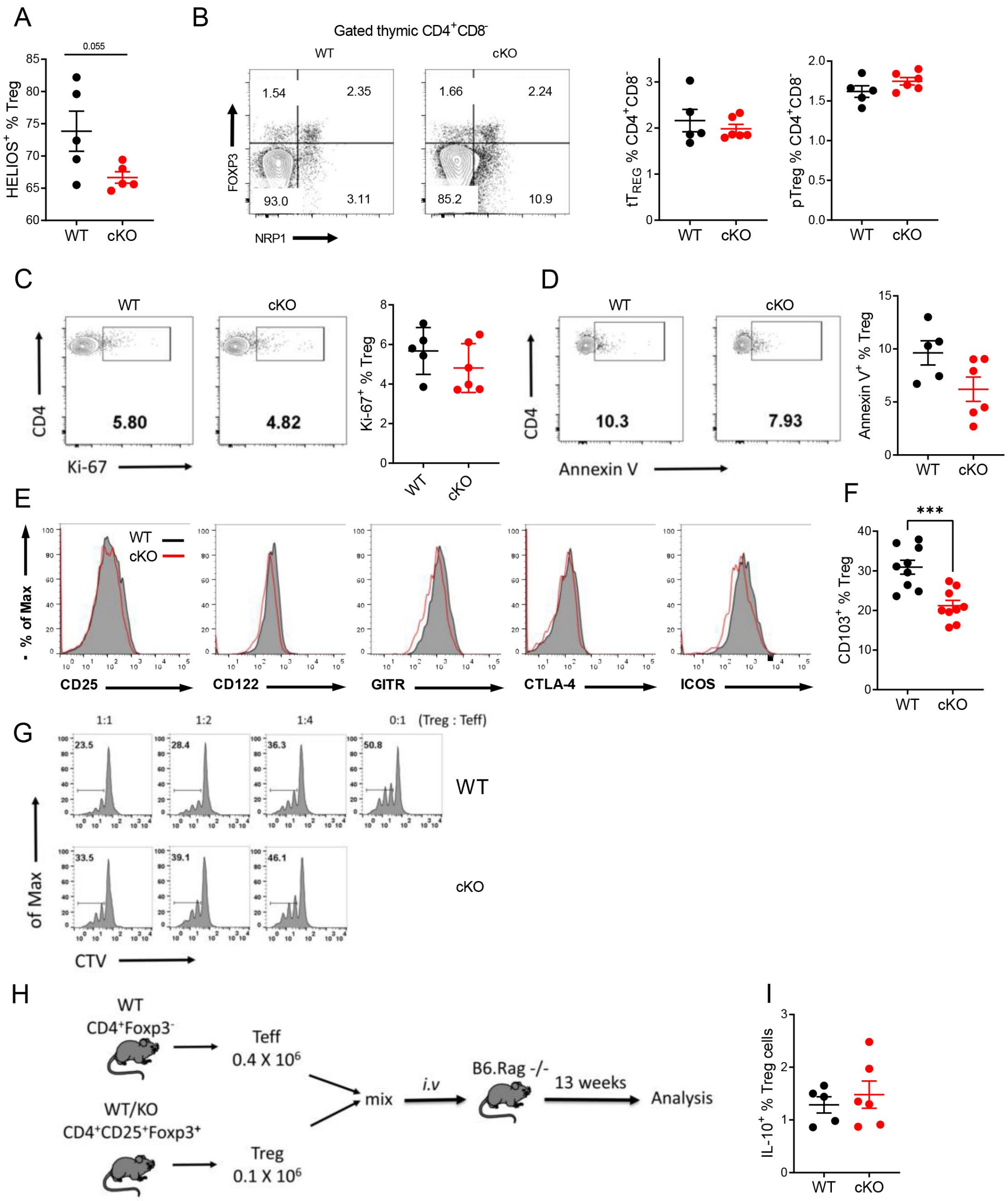

Figure S3. Effect of *Esrrg* deficiency on thymic Treg cells, Treg proliferation, apoptosis and differentiation. (**A**) Frequency of splenic HELIOS$^+$ Treg cells. (**B**) Representative CD4$^+$CD8$^-$-gated FACS plots showing thymus-derived NRP1$^+$ tTreg cells and peripheral NRP1$^-$ pTreg in thymus from WT and cKO mice. Representative CD4$^+$FOXP3$^+$-gated FACS plots showing Ki-67$^+$ Treg cells (**C**) and Annexin V$^+$ Treg cells (**D**) from WT and cKO spleens, and corresponding quantitations. (**E**) Representative CD4$^+$FOXP3$^+$-gated histograms showing the expression of CD25, CD122, GITR, CTLA-4 and ICOS. (**F**) Frequency of CD103 expression on splenic Treg cells. (**G**) Representative CD4$^+$CD45.1$^+$-gated FACS histograms showing the percentage of proliferating Teff cells with increasing CD45.2$^+$-Treg/ CD45.1$^+$-Teff ratios. **H**. Experimental design showing WT Teff cells co-transferred with WT or cKO Treg cells into B6.Rag$^{-/-}$ mice. **I.** Frequency of IL-10$^+$ Treg cells from WT and cKO spleens. Data are shown as mean ± SEM. Unpaired *t*-tests, *** *P* < 0.001.

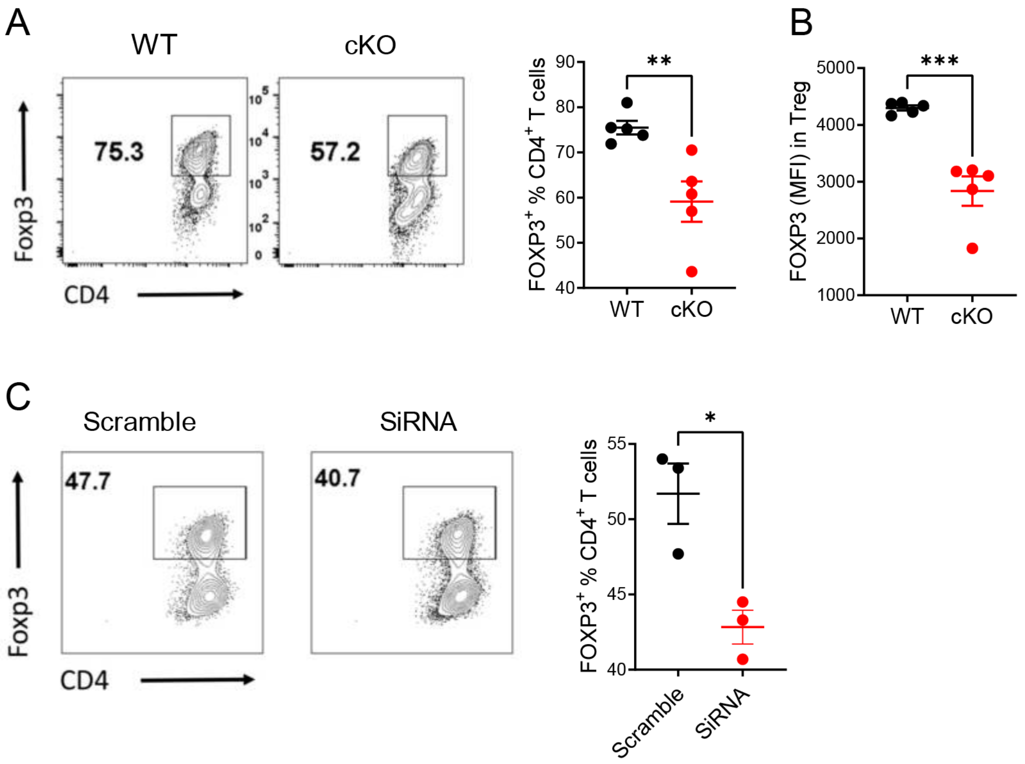

Figure S4. *Esrrg* regulates Treg cell polarization. A) Representative $CD4^+$-gated FACS plots showing the frequency of $FOXP3^+$ cells differentiated from $CD4^+CD25^-$ T cells from cKO and WT mice under Treg polarizing conditions for 3 d, with quantification on the right. (**B**) FOXP3 expression in iTreg cells. (**C**) $CD4^+CD25^-$ T cells from B6N mice were stimulated with anti-CD3/CD28 for 2 d with/without *Esrrg*-siRNA, then switched to Treg polarizing conditions for an additional 3 d. Representative $CD4^+$-gated FACS plots showing the frequency of $FOXP3^+$ cells, with quantification on the right. Data are shown as mean ± SEM. Unpaired *t*-tests, * $P < 0.05$, ** $P < 0.01$, *** $P < 0.001$.

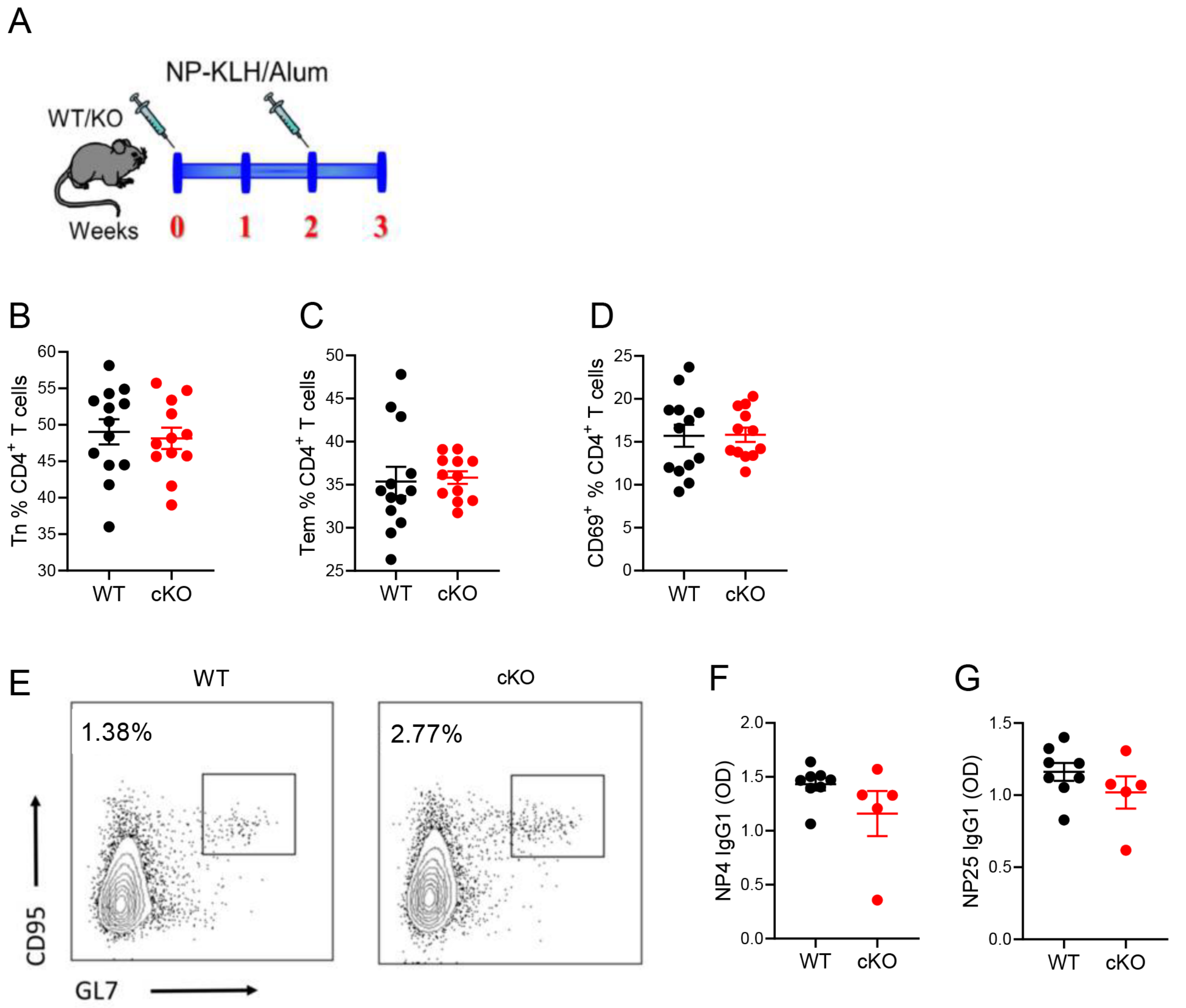

Figure S5. T cell subsets and antibody production after NP-KLH immunization. (**A**) Experimental design with analysis at week 3. Percentage of naïve (Tn) (**B**), effector memory cells (Tem) (**C**), and CD69$^+$ (**C**) in CD4$^+$ T cells. (**E**) Representative B220$^+$-gated FACS plots showing CD95$^+$GL7$^+$ GC B cells. Serum anti-NP-specific IgG1 were measured against NP4 (high affinity) and NP25 (low affinity). Data are combined from three independent experiments (**B** - **D**) and antibody production was from one of three independent experiments (**F** - **G**). Data are shown as mean ± SEM, unpaired *t*-tests.

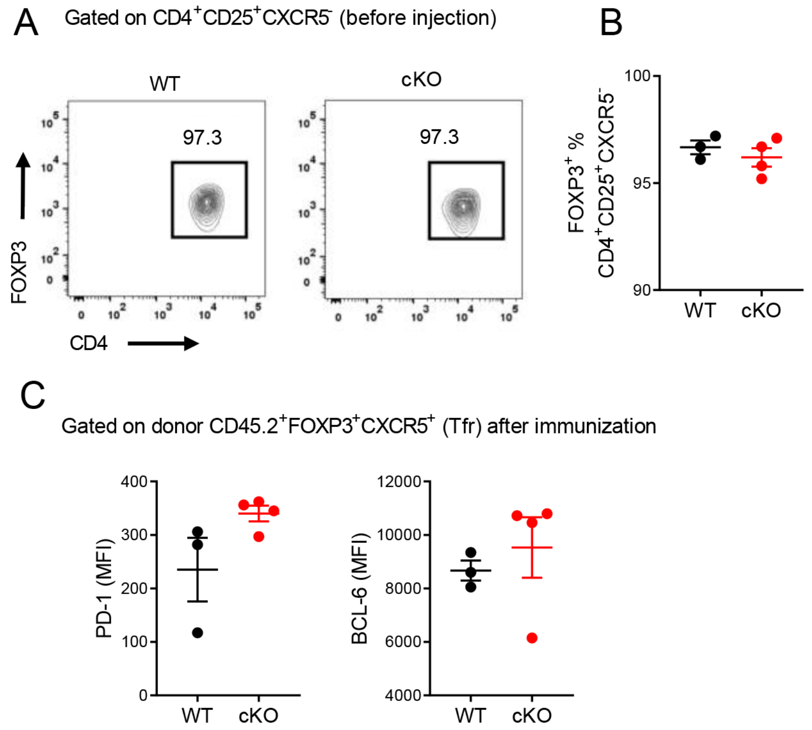

Figure S6. *Esrrg* deficiency in Treg cells impairs the formation of Tfr cells. (**A**) Representative CD4^{+}-gated FACS plots showing the percentage of Foxp3^{+} Treg cells in sorted CD4^{+}CD25^{+}CXCR5^{-} cells from B6N.*Foxp3$^{YFP\text{-}Cre}$* (WT) or B6N.*Esrrg$^{f/f}$ Foxp3$^{YFP\text{-}Cre}$* (cKO) mice that were injected into B6.SJL mice. (**B**) Quantification of FOXP3^{+} Treg cells in (**A**). (**C**) Expression of PD-1 and BCL-6 in Tfr cells from WT or cKO mice in immunized B6.SJL mice. Data are presented from one of two independent experiments, unpaired *t*-tests.

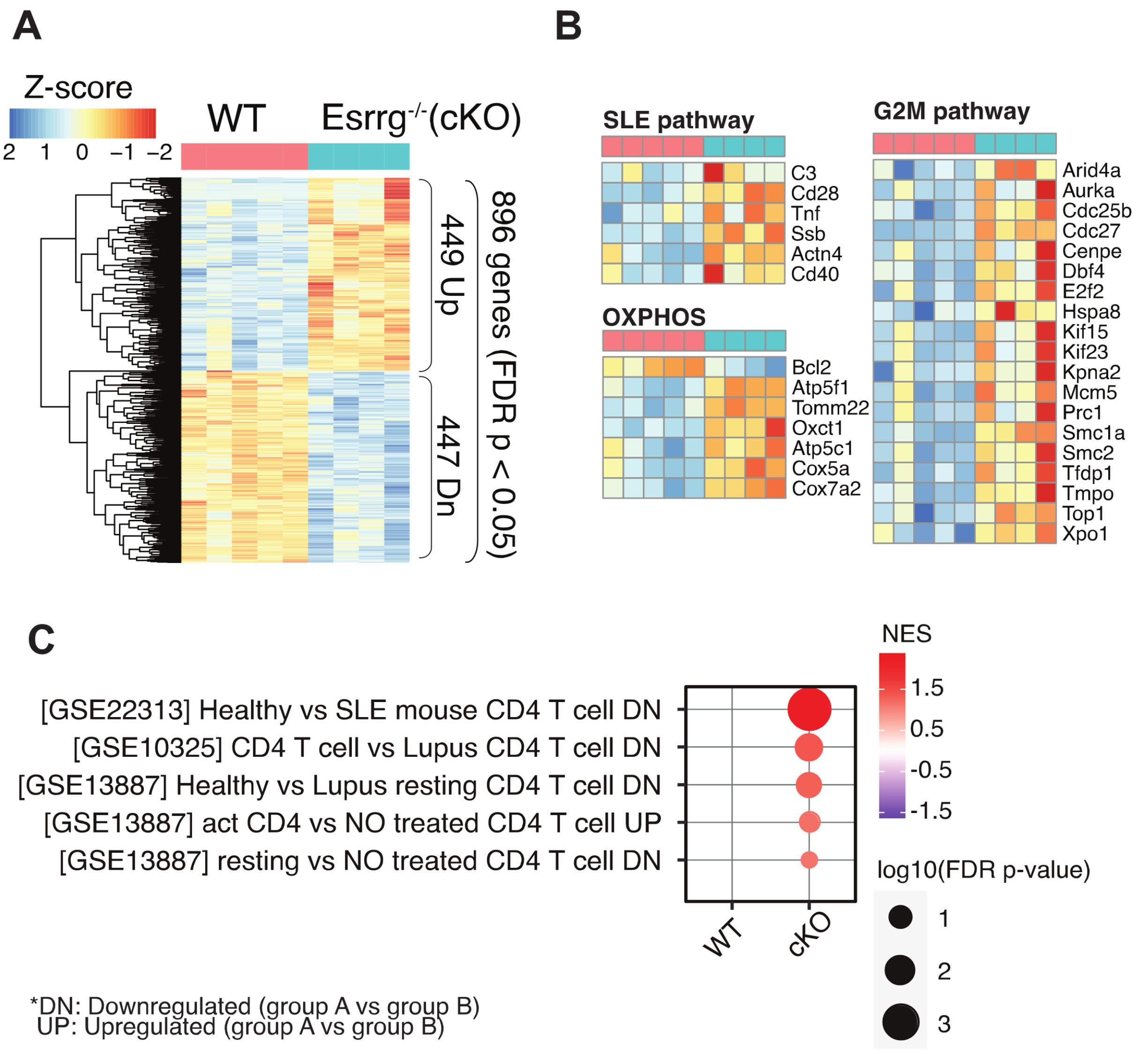

Figure S7. Transcriptomic programming of Treg by *Esrrg.* (**A**) RNA-seq analysis was performed on CD4⁺Foxp3⁺ Treg cells from B6N.*Foxp3*$^{YFP\text{-}Cre}$ (WT) and B6N.*Esrrg*$^{f/f}$.*Foxp3*$^{YFP\text{-}Cre}$ (cKO) mice. The heatmap shows the 449 upregulated and 447 downregulated genes in cKO Treg cells. (**B**) Heatmap showing a selection of differentially expressed genes in the SLE, G2M and OXPHOS pathway. (**C**) GSEA analysis comparing the ranked gene list of *Esrrg*-cKO Treg cell RNA-seq profiles with other publically available SLE-related CD4⁺ T cell gene sets.

Table S1. Primers used in this paper.

**Human**	
Primer name (5' – 3')	
HMBS-F	AGAATGAAGTGGACCTGGTTGT
HMBS-R	AGATGGCTCCGATGGTGAAG
ESRRG-F	CTGAAAGAAGGGGTGCGTCT
ESRRG-R	GGCTTTTTGGCTGGCTGAAC
**Mouse**	
Primer name (5' – 3')	
Gzma-F	CCACTGTAACGTGGGAAAGAGA
Gzma-R	AGTTGTAGATCCCCCTCACG
Cdc27-F	CACAGTTGTACCACCCCACA
Cdc27-R	CTGGCTTCTCACCAATACACA
Gzmb-F	TGCTAAAGCTGAAGAGTAAGGC
Gzmb-R	AGGACTCACACTCCCGATCC
Smurf2-F	AGCATTATGACCTGTATATCGGAA
Smurf2-R	TAACCAGTGTCCTTGAGGCG
Smad4-F	ACGACTTTGAAGGACAGCCG
Smad4-R	TACTCAGGAGTTGTGGAAGCC
Il2ra-F	TGGCAACACAGATGGAGGAA
Il2ra-R	CGTTAGGTGAATGCTTGGCG
Hmbs-F	AGATGGGCAACTGTACCTGAC
Hmbs-R	GGATGGTGGCCTGCATAGTC
Ppia-F	GCTGTTTGCAGACAAAGTTCCA
Ppia-R	CGTGTAAAGTCACCACCCTGG
**FANA iRNA oligo**	
Esrrg-1	TAACCACCAACTCTCGGTC
Esrrg-2	AGAGCTATAGCTTTGAGGG
